# HIV Promoter Integration Site Primarily Modulates Transcriptional Burst Size Rather Than Frequency

**DOI:** 10.1371/journal.pcbi.1000952

**Published:** 2010-09-30

**Authors:** Ron Skupsky, John C. Burnett, Jonathan E. Foley, David V. Schaffer, Adam P. Arkin

**Affiliations:** 1 California Institute for Quantitative Biosciences, University of California, Berkeley, Berkeley, California, United States of America; 2 Department of Chemical Engineering, University of California, Berkeley, Berkeley, California, United States of America; 3 Helen Wills Neuroscience Institute, University of California, Berkeley, Berkeley, California, United States of America; 4 UCB/UCSF Joint-Graduate-Group-in-Bioengineering, University of California, Berkeley, Berkeley, California, United States of America; 5 Department of Bioengineering, University of California, Berkeley, Berkeley, California, United States of America; 6 Physical Biosciences Division, Lawrence Berkeley National Laboratory, Berkeley, California, United States of America; Weizmann Institute of Science, Israel

## Abstract

Mammalian gene expression patterns, and their variability across populations of cells, are regulated by factors specific to each gene in concert with its surrounding cellular and genomic environment. Lentiviruses such as HIV integrate their genomes into semi-random genomic locations in the cells they infect, and the resulting viral gene expression provides a natural system to dissect the contributions of genomic environment to transcriptional regulation. Previously, we showed that expression heterogeneity and its modulation by specific host factors at HIV integration sites are key determinants of infected-cell fate and a possible source of latent infections. Here, we assess the integration context dependence of expression heterogeneity from diverse single integrations of a HIV-promoter/GFP-reporter cassette in Jurkat T-cells. Systematically fitting a stochastic model of gene expression to our data reveals an underlying transcriptional dynamic, by which multiple transcripts are produced during short, infrequent bursts, that quantitatively accounts for the wide, highly skewed protein expression distributions observed in each of our clonal cell populations. Interestingly, we find that the size of transcriptional bursts is the primary systematic covariate over integration sites, varying from a few to tens of transcripts across integration sites, and correlating well with mean expression. In contrast, burst frequencies are scattered about a typical value of several per cell-division time and demonstrate little correlation with the clonal means. This pattern of modulation generates consistently noisy distributions over the sampled integration positions, with large expression variability relative to the mean maintained even for the most productive integrations, and could contribute to specifying heterogeneous, integration-site-dependent viral production patterns in HIV-infected cells. Genomic environment thus emerges as a significant control parameter for gene expression variation that may contribute to structuring mammalian genomes, as well as be exploited for survival by integrating viruses.

## Author Summary

Cellular gene expression is a fundamentally stochastic process due to the intrinsic randomness of the underlying biochemical reactions involved. The resulting stochastically generated expression heterogeneities have important biological consequences and also encode information about the underlying dynamics that generate them. A fundamental goal of transcriptional biology is to understand the quantitative regulation of gene-expression dynamics, which in eukaryotes depends on factors specific to each gene in concert with its surrounding cellular and genomic environment. We investigated the regulatory effects of variable genomic environments by quantitatively measuring expression heterogeneity from diverse single genomic integrations of the HIV promoter in cultured cells. Systematically fitting a model of stochastic gene expression to our measurements reveals transcript production in bursts as the underlying dynamic that accounts for the large heterogeneities observed within single-integration clonal cell populations, with the size of transcriptional bursts as the primary feature that varies over genomic integrations. Our findings implicate genomic environment as an important quantitative control parameter that eukaryotic cells might use to shape gene-expression patterns, and that lentviruses such as HIV, whose genomes are semi-randomly integrated into the genomes of the host cells they infect, may exploit to sample diverse and heterogeneous expression patterns that evade treatment.

## Introduction

The life cycle dynamics of HIV-1 within a host are shaped by numerous apparently stochastic processes, from the statistics of immune cell infection in humans, to mutation during reverse transcription, semi-random integration of the proviral DNA into the host-cell chromosome, and stochastic viral gene expression thereafter [1]–[7]. We and others have experimentally shown that expression from the HIV-1 promoter is indeed stochastic and shaped by host factors at the viral integration site [4], [8], [9], [10], and we have argued as well that the resultant expression heterogeneities are important in the genesis of viral latency [4], a ubiquitous feature of infection that currently confounds our ability to cure HIV in patients [7], [11], [12], [13]. Gaining a deeper understanding of the factors that influence cell-cell variability in viral gene expression may thus shed light on how to ameliorate the effects of latency, and more generally on the processes that affect the expression of any gene.

The semi-random integration of HIV-1 into the host genome provides a particularly ideal opportunity to dissect the relative contribution of genomic environment as a fundamental element of expression regulation that may contribute importantly to expression dynamics and heterogeneities in eukaryotes. It is now well established that HIV-1 integration is biased towards actively transcribed chromosomal locations [3], [14], and it has been demonstrated that mean expression levels from model HIV-1 viruses correlate with specific epigenetic features at their integrations [9] and of their surrounding genomic regions [3]. Prior studies in other systems focused on how the population average expression of genetic constructs depends on integration context, and have found correlations with the expression levels of surrounding genes and with the local 3-D chromatin structure [15], as well as with DNA methylation, nucleolar association, and DNA diffusional mobility [16]. Importantly, these studies inform us about the features of genomic environment that might affect mean expression levels. However, the effects of genomic environment, or integration site, on stochastic expression and heterogeneity have not yet been explored.

The discrete and stochastic nature of gene expression has been appreciated for some time [17], [18], [19], and it has become increasingly recognized that the resulting expression variability may significantly impact diverse biological functions, shaping the outcomes of cellular decisions, being exploited as a tool for survival in changing environments, and often inducing qualitatively different behaviors than would be predicted from a deterministic understanding [20]–[26]. Theoretical and computational analyses have explored the relative contributions of key processes to heterogeneity in gene expression, including open-complex formation, transcriptional elongation, translation, post-transcriptional and translational processing/modification, as well as chromatin regulation [27]–[32]. Importantly for this study, the latter, an integral element of epigenetic control over gene expression, yields potentially slow and probabilistic dynamics that have been postulated as a significant source of expression heterogeneity in eukaryotes. In parallel with these theoretical studies, experimental approaches have been developed to characterize expression noise arising both ‘intrinsically’ from the biochemical processes directly involved in the expression of any individual gene, as well as ‘extrinsically’ from variability in other cellular processes that more homogeneously affect the expression dynamics of groups of genes simultaneously, such as cell cycle or concentration fluctuations of upstream transcription factors [33]–[37]. Interestingly, genome-scale measurements of expression heterogeneity demonstrate correlations with gene functional class, implying that perhaps noise is a “selected” feature of a gene's expression pattern [38]–[43].

Despite the apparent complexity of cellular transcriptional regulation, for many genes across a broad range of cell types, the patterns of cell-to-cell expression variability within isogenic populations are remarkably well described by simple stochastic models that represent the gene – including the associated genomic environment, chromatin structure, transcriptional regulators, and transcriptional machinery – as existing in a small number of discrete configurations, or states, with expression heterogeneities depending on probabilistic transitions between states and on probabilistic transcript and protein production and degradation [27], [44]. These models are often necessarily abstract, yet they parsimoniously capture many essential features of transcriptional biology. Model fits to clonal single-cell experimental data, primarily in *Saccharomyces cerevisiae* for eukaryotic studies, has allowed the inference of underlying gene-state and transcriptional dynamics that largely account for observed expression heterogeneities in a number of instances [39], [45]–[49].

A wide range of transcriptional dynamics have been revealed by such analyses, from continuous [38], [48], to ‘pulsatile’ [45], [47], to ‘bursty’ [46], [48]. These diverse dynamics are effectively characterized by the frequency of gene-activation events, their duration, and the number of transcripts produced during each event [29], [30], [48], [50], and contrasting results have emerged concerning the relative contributions of cellular regulation of each of these quantities to specifying the expression pattern of any given gene. For example, several pioneering studies, of single, targeted integrations of inducible, synthetic constructs, in yeast have suggested that the concentration of inducer largely controls the frequency of gene-activation events rather than the number of transcripts produced by each event [45], [47]. A subsequent study in yeast – which considered three targeted integrations of similar constructs into 1) adjacent locations on a single chromosome, 2) homologous locations on sister chromosomes, or 3) non-homologous chromosomal locations – similarly found that transcriptional activation frequency varied between locations [36]. Genome-scale studies of stochastic gene expression in yeast suggest as well that the primary feature of transcriptional dynamics that varies between genes, over a wide range of genes, is the frequency of transcriptional activation events [38], [39]. In contrast, an elegant study in mammalian cells quantified expression heterogeneities – from a single, random integration of a Tet-inducible construct into one locus in the genome – by using fluorescent in-situ hybridization (FISH) to directly visualize single transcripts [46]. The authors concluded that transcripts are produced in bursts, and that the typical number of transcripts produced during a burst (referred to as the ‘transcriptional burst size’), rather than the frequency of bursting, was the primary measure that varied with tetracycline induction level.

While the above studies have begun to characterize the dependence of gene-expression dynamics on a number of cellular inputs, a systematic, quantitative investigation of the contribution of genomic environment over a broad range of genomic integration positions remains to be conducted. Furthermore, the contrasting observations as to whether transcriptional activation frequency, transcriptional burst size, or some other feature of transcriptional dynamics represents the primary variable that cells modulate to control expression patterns in these diverse systems raise key questions of how important features of genetic, epigenetic, and regulatory architecture may differ in yeast and mammalian cells.

Here we explore the fundamental relationship between genomic environment and expression heterogeneity from a diverse set of semi-random single integrations of a model HIV-1-promoter/GFP-reporter construct in cultured Jurkat T-cells. Systematically and rigorously fitting a model of stochastic gene expression allows us to infer the underlying expression dynamics that account for the single-gene expression distributions that we measure from single-integration clonal populations. Our analysis reveals that transcript production in bursts accounts for the wide, highly skewed, expression profiles that we observe, and importantly that transcriptional burst size is the primary feature that varies across viral integrations. These results interestingly suggest that the virus samples a particularly ‘noisy’ range of possible expression profiles across cellular integrations, and open a number of important questions for further study. We propose several qualitative models that may explain this inferred variation of transcriptional dynamics with genomic environment and discuss the implications of our findings for HIV dynamics, and for cellular gene expression in general.

## Results

### HIV-1 LTR distributions are wide and highly skewed

**Figure 1:**
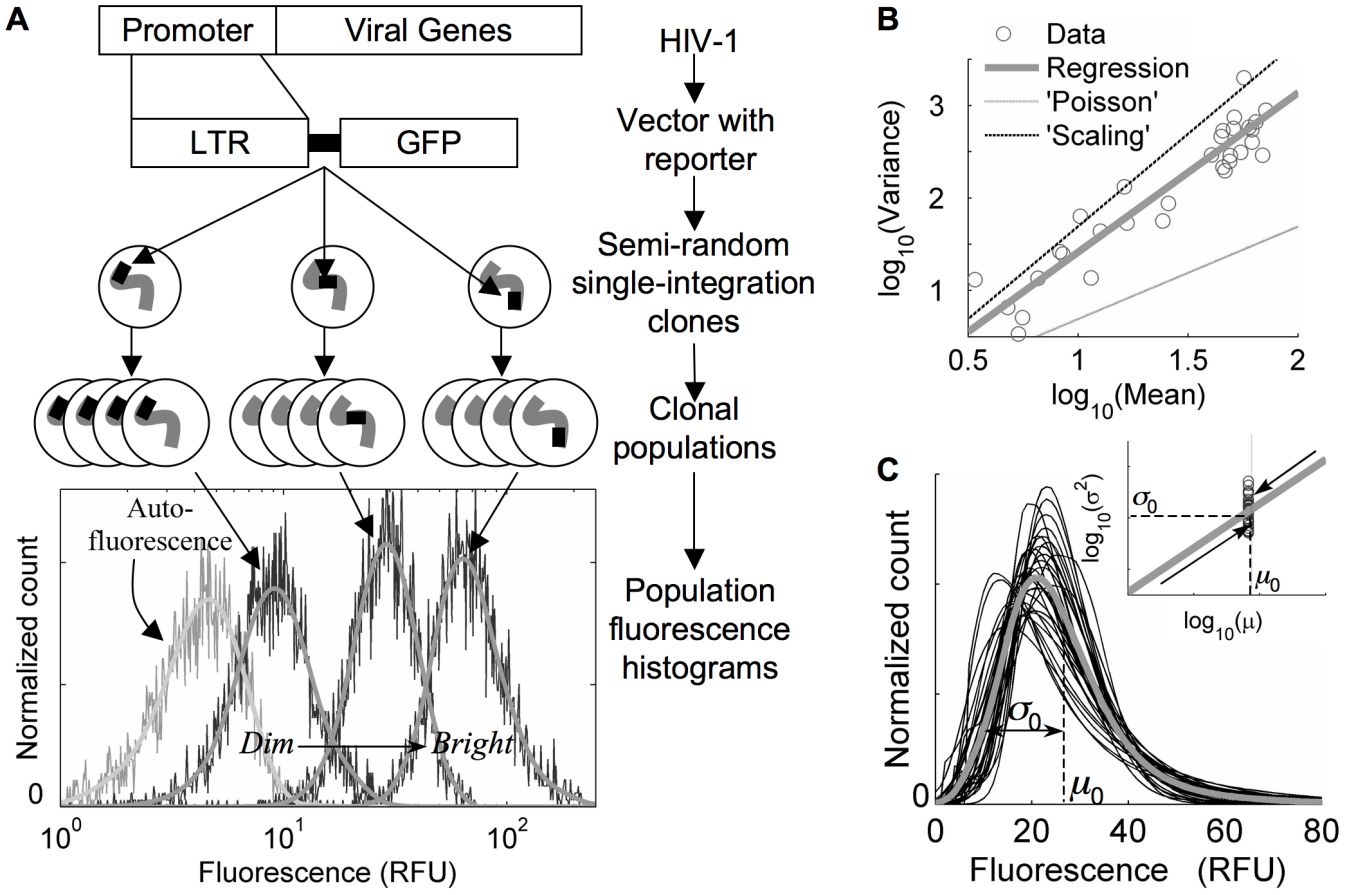
HIV LTR expression distributions are wide and highly skewed. **A**) **Experimental system**: Sample clonal histograms, spanning a range of ‘dim’ and ‘bright’ integrations, with autofluorescence included for comparison, represent fluorescence measurements on approximately 4000 cells. Smooth curves through each are the result of an optimized low-pass Fourier filter, and are used for model fitting. All measurements are given in cytometer-based relative fluorescent units (RFU). **B**) **Trend in distribution-shape variation**: Log-log linear regression coefficients quantify trends in distribution-shape variation as a power-law relationship between distribution variance (σ^2^) and mean (μ): 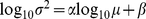; 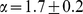 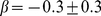 (*R^2^* = 0.89, coefficients ±95% confidence). Dashed lines demonstrate Poisson-like scaling (‘Poisson’, α = 1) and over-all distribution scaling (‘Scaling’, α = 2). **C**) **Characteristic distribution shape**: Smoothed, autoflorescence-deconvolved histograms were shifted by a constant fluorescence to a fixed mean (specified by the median over the set of integration clones, *μ_0_*), and fluorescence values were scaled about that mean according to the variance regression in 1B (inset), by a factor 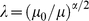 with α = 1.7 (thin curves). The grey curve averages the transformed distributions and represents a ‘typical’ HIV-LTR expression profile that is wide (coefficient of variation = σ_0_/*μ_0_*∼60%) and highly skewed.

Although HIV-1 requires transactivation by the virally-encoded protein Tat to amplify its expression [51], the HIV-1 promoter still supports basal transcription in the absence of Tat [9], which occurs initially after viral infection but before significant viral protein is produced. The dynamics of this basal expression, and the associated expression heterogeneities that result, may play an important role in affecting the cellular ‘decision’ between lytic viral production and latency [4]. To study heterogeneities in basal expression from the HIV promoter, we infected Jurkat T-cells, at a low multiplicity of infection (MOI), with a model HIV-1 virus that contains the full-length LTR driving expression of a GFP reporter but no viral genes. Cells with single integrations were isolated by fluorescence activated cell sorting (FACS) and expanded into clonal populations. The resulting clonal GFP expression profiles were quantified by flow cytometry and smoothed for comparison to model distributions in the analysis that follows. Thirty-one such clones, with average florescence levels ranging over an order of magnitude, and expression profiles clearly distinguishable from a measured autofluorescence profile, were selected for analysis (Fig. 1A). Integrations whose mean fluorescence was less than twice the autofluorescence mean were not included in our analysis.

The shape features of our experimental distributions (such as mean, variance, skewness, etc.) are diagnostic of the underlying expression dynamics that generate them – and of the regulatory role of various molecular ‘inputs’ such as integration position (as well as promoter structure and concentrations of transcription factors, which were the ‘inputs’ considered in several other elegant studies: [38], [45], [46], [47]). For example, a simple model assuming transcript number fluctuations as the primary source of expression heterogeneity, with only the rate of constant transcript production varying with a given ‘input’, predicts a Poisson-like distribution shape variation whereby distribution variances (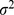 considered as a measure of distribution width and expression heterogeneity) vary proportionately to the mean (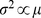, for mean 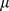). Such a variation, illustrated by the lower dashed line in Fig. 1B (‘Poisson’), has been observed over a large set of yeast promoters under multiple experimental conditions [38], [39]. Alternatively, a model in which distribution shape variations are effectively described by a simple scaling of single-cell fluorescent values by an ‘input-controlled’ constant value (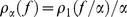, where 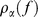 is the probability of observing fluorescence 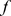, for a normalized value of the ‘input-controlled’ parameter 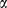) would predict distribution variances to vary in proportion to the mean *squared* (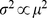, Fig. 1B upper dashed line, ‘Scaling’). Such a shape variation might arise if heterogeneities are instead due primarily to probabilistic transitions between promoter configurations that specify different transcription rates, with only these transcription rates varying (proportionately) with the ‘input’ from one clonal distribution to the next. In contrast to these possibilities, we find that the trend in distribution-shape variation over our set of clonal populations is best described by a relationship where the distribution variance changes proportionately to the mean raised to the 1.7±0.2 power (Fig. 1B, solid regression). This characteristic trend differs significantly from either of the above simple models (*P*<0.025), suggesting that neither is sufficient, and that integration-site variation may specify a more complex modulation of promoter and transcriptional dynamics in our system.

To visualize additional features of the expression distributions over the set of clones, we translated each to a common mean fluorescence, and correspondingly scaled its fluorescence values about that mean based on the variance regression in Fig. 1B, revealing a ‘typical’ distribution shape that is wide and highly skewed (Fig. 1C). These features are signatures of a bursty underlying transcriptional dynamic [27], [50], as we discuss in further depth below.

### A two-state gene model of transcriptional bursting *qualitatively* captures characteristic HIV-LTR distribution shapes and variation over viral integrations

**Figure 2:**
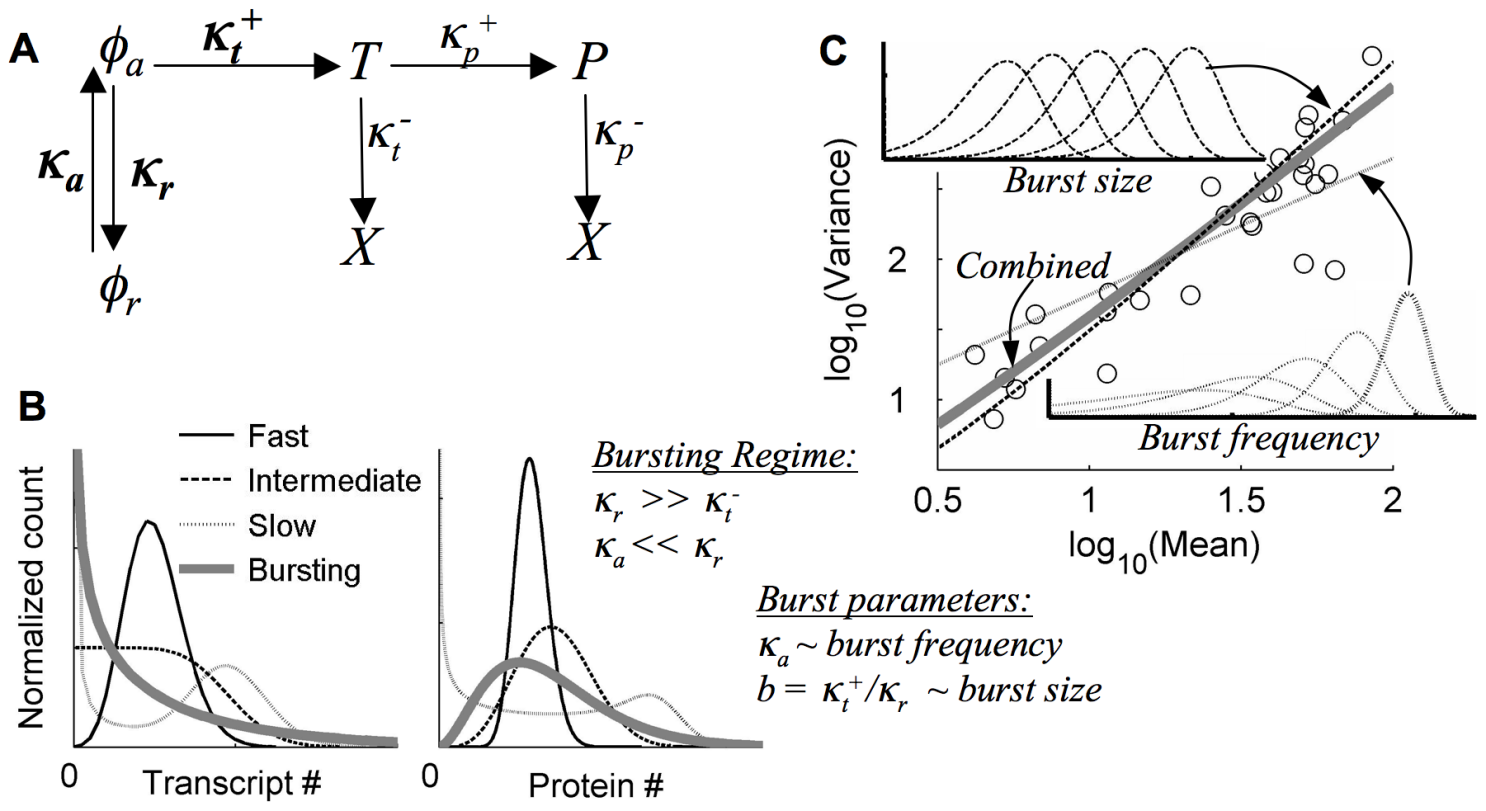
Transcript production in bursts qualitatively explains HIV LTR distribution shapes and variation with integration site. **A**) **Model schematic**. *φ_a/r_* = active/repressed gene state, *T* = Transcript, *P* = Protein, *X* = Degraded, κ = Probability/unit time. Bold parameters are considered to be integration-site dependent, while others were measured separately and fixed for all clones. All rates are measured relative to the transcript degradation rate, *κ_t_^−^*. **B**) **Model regimes** depend on the ratio of gene-state to transcriptional dynamics: ‘Fast’ (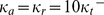), ‘Slow’ (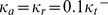), ‘Intermediate’ (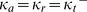), ‘Bursting’ (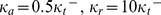). Transcript production rates (*κ_t_^+^*) for sample distributions are set to reproduce the same mean number of transcript copies (left) and protein copies (right) at steady state as predicted for the ‘typical’ experimental distribution in Fig. 1C; autoflourescence is not included, and distributions are binned on a linear RFU scale for comparison. **C**) **Distribution shape-variation in the bursting regime**: burst-frequency variation (*κ_a_*) leads to an approximate Poisson-like shape-variation, burst-size (*b*) yields an approximate distribution-scaling shape variation, and the combination with 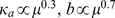 (combined) gives a shape-variation most closely resembling the experimental data (compare Fig. 1B). Insets are sample, log-binned distributions with varying burst size or frequency. The fixed parameter in each sample is set to the value that approximately reproduces the ‘typical’ distribution shape in Fig. 1C.

A simple stochastic model that captures a number of essential features of transcriptional biology, and that can reproduce a range of single-gene expression profiles, assumes that the promoter may exist in either an activated state (*φ_a_*) that produces mRNA probabilistically at a fixed rate (*κ_t_^+^*), or repressed state (*φ_r_*) that is unproductive (Fig. 2A). These model states may represent different characteristic configurations of chromatin and/or transcriptional complexes, with transitions between them occurring at rates *κ_a_* and *κ_r_*. Together with the active-state transcription rate, these lumped parameters represent contributions from diverse modes of genetic and epigenetic transcriptional regulation that may depend essentially on features of the genomic environment at the viral integration sites. Variants of this model have been used in other studies as well to analyze single-gene expression data [45]–[49] and have also been studied theoretically [27], [52], [53], [54].

In the analysis that follows, we always consider steady-state model distributions, since longitudinal measurements over the course of a week on several clones indicate that distribution shapes are relatively stable over our time scale of interest (see Fig. S3 and Sec. S.VII for further discussion). Furthermore, we determine all rate constants relative to the transcript degradation rate (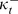, estimated to be approximately 0.2 h^−1^, see Text S1 Sec. S.V), as their relative rather than absolute values determine expression profiles at steady state. In addition, we adopt the working hypothesis that our experimental distribution shapes are determined by the *intrinsic* processes represented in our model at *fixed* values of its rates – possible contributions of extrinsic sources of variability have been considered in earlier work on this system [4] and are discussed further in the Supporting Information (Text S1, Sec. S.VIII).

The qualitative expression regimes of the two-state gene model fundamentally depend on the relative values of the gene-state transition rate constants (Fig. 2B), with different dynamics corresponding to different potential underlying transcriptional regulatory mechanisms. ‘Fast’ gene-state dynamics (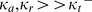, perhaps specified by fast binding and unbinding of transcription factors) approximate continuous transcription from a single fixed gene state and can generate relatively narrow Poisson-like expression profiles, which widen for ‘Intermediate’ dynamics (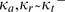). ‘Slow’ gene-state dynamics (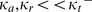, due perhaps to slower changes in chromatin configuration) may generate multiple transcripts after each transition to a relatively stable active state, and the dynamics can be described as ‘pulsatile’ [45], [47]. Distributions become bimodal in the extreme case.

Another dynamic regime that has received considerable attention can be termed the transcriptional ‘bursting’ regime, in which the gene inactivation rate is fast (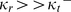), and the transcription rate is sufficiently large (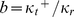 not small) that transcriptional bursts of average size 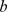 are produced during short excursions of frequency 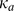 to a relatively unstable active gene-state (see Text S1, Sec. S.V for further discussion, and Refs. [29], [30], [48], [50], [55]). Distributions in the ‘bursting’ regime are wide and highly skewed, in qualitative agreement with the ‘typical’ HIV-LTR distributions from our measurements (compare Fig. 1C and 2B, solid curves), with both the protein and transcript distribution means and variances approximately demonstrating a relatively simple dependence on transcriptional burst size and frequency: 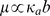; 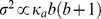 (see Text S1, Secs. S.III – S.V). Indeed, by assuming a model solution in the ‘bursting’ regime, one can analytically calculate a unique transcriptional burst size and burst frequency that reproduce the mean and variance of each of our experimental distributions, with good *qualitative* agreement in distribution shape (see Text S1, Secs. S.III and S.VI for further discussion). Furthermore, variation in transcriptional burst size and frequency, individually or in combination, can account for the range of distribution-shape variation discussed in Fig. 1B, with the best agreement to our experimental observations occurring if burst size and burst frequency typically vary simultaneously, but with the dominant effect coming from burst-size variation (Fig. 2C).

Though the relatively slow time scale of protein degradation in our system (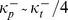) effectively ‘filters’ some of the dynamic information propagated from model transcript to protein distributions, we emphasize that the calculated protein distribution shapes still reflect the underlying transcript distribution shapes and demonstrate distinctive features in each expression regime (Fig. 2B). Below, through careful analysis, we will make use of this observation, building on the qualitative analysis developed here, to quantitatively infer the underlying heterogeneity-generating gene-state and transcriptional dynamics within our system from measured protein expression distributions, and to determine quantitative bounds on our ability to distinguish between different dynamic regimes. Of considerable benefit for this analysis, cytometry-based protein measurements can be acquired rapidly (e.g. compared to microscopy-based transcript-counting measurements), allowing good resolution of the probability distributions that underlie the expression histograms collected over populations of cells, and enabling measurements on sufficient numbers of clones to identify trends in the variation of single-gene expression distributions over integration sites.

### Bursting gene expression *quantitatively* accounts for HIV-1 LTR integration-clonal distributions

While the analysis above provides intuition as to the dynamics and regulation that may underlie our experimental observations of the HIV LTR, it is solely a qualitative assessment based on the assumption of ‘burstiness’ and comparisons to ‘stereotypical’ model distributions. In reality, model distributions vary continuously between regimes, and means and variances provide an incomplete characterization of the actual distribution shapes. Therefore, to better determine the degree to which transcriptional ‘bursting’ best accounts for our experimental distributions, and the degree to which it can be distinguished from other possible dynamic regimes, we used a systematic fitting routine to identify the best-fit combination of transcription rate and gene-state transition rates for each distribution. Transcript degradation, protein production, and protein degradation rates (*κ_t_* ^−^, *κ_p_* ^+^, and *κ_p_* ^−^, Fig. 2A) were fixed at values that were separately measured.

An important indicator of the dynamic regime of our system is the average time that the promoter remains in the active configuration following a gene activation event, relative to the average life time of a transcript (see Fig. 2B), specified by τ = *κ_t_^−^*/*κ_r_*, which we refer to as the ‘active duration’ (τ). We therefore began by identifying best-fit sets of model parameters for each clone over a range of fixed values for τ. We arrived at a robust estimate of the range of parameters for which the model quantitatively accounts for our measured distributions by considering the ratio, *Dev_r_*, of each best-fit deviation at a given τ, to a bootstrap-estimated 95% upper bound on the deviation expected due to uncertainty in our measurements, which served as a metric for identifying model fits whose quality was statistically comparable. Fits for which the values of *Dev_r_* differ by less than 1 for a given clone were considered to be effectively indistinguishable, since their differences may be accounted for by uncertainty in our experimental data, and these fits were thus considered to identify a range of parameters for which the model gives a statistically comparable account. The work-flow for our analysis is summarized in Table S1; the definitions that we used to quantify fit deviations, as well as the error model used for our bootstrap error calculation, are discussed briefly in the Materials and Methods, and in more depth, Text S1, Secs. S.I and S.VII, together with Fig. S1.

**Figure 3:**
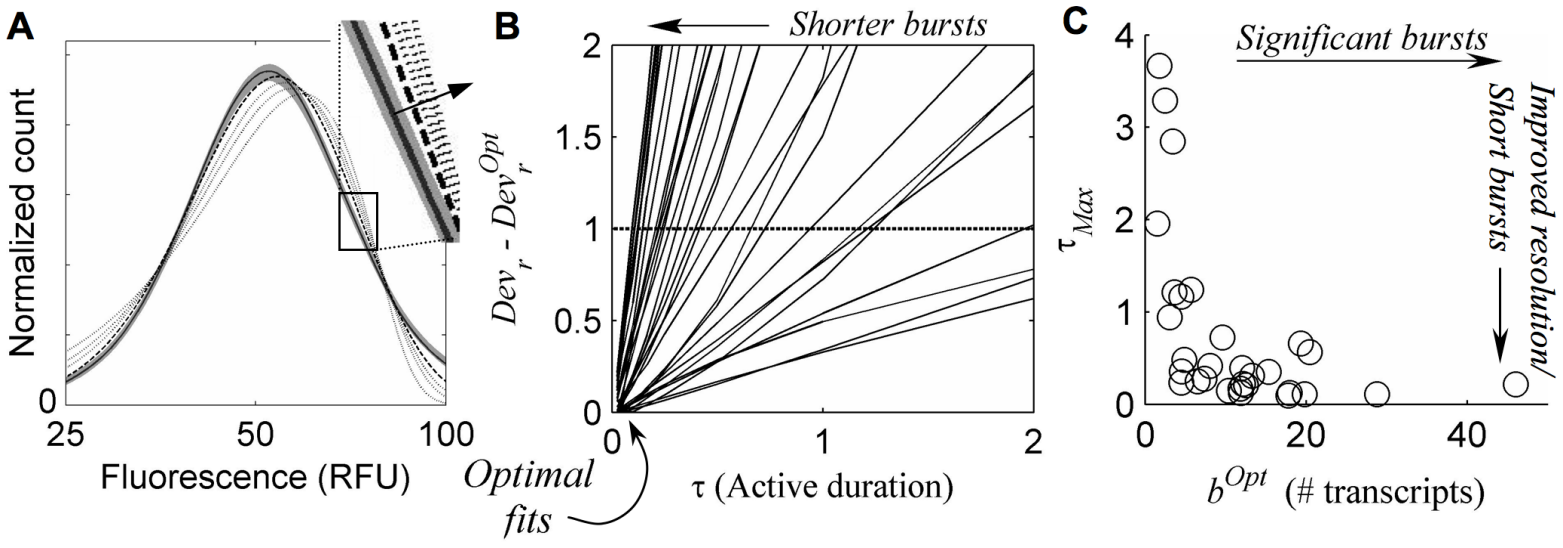
Transcriptional bursts are short, but only an upper bound on their duration can be resolved. **A**) **Sample fits** for several fixed values of burst duration, τ (measured relative to the transcript degradation time): experimental distribution (solid curve with 95% confidence region in grey); optimal fit at small τ (long dash); fits for larger 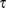 (short dash), demonstrating increased deviations (τ increasing along arrow in for inset). **B**) **Relative fit deviation,** ***Dev_r_*** **decreases for shorter active duration τ**, for each clone (solid lines), and are optimal (*Dev_r_* = *Dev_r_^Opt^*) when burst durations are shortest (i.e. in the bursting regime). *Dev_r_*−*Dev_r_^Opt^* = 1 (dashed line) is considered a cut-off, beyond which fit quality is significantly worse than the optimum, specifying a distinguishability cut-off upper-bound on τ, marked by the intersection of dashed line and the solid lines and referred to as 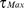 for each clone. **C**) **Resolution of bursting dynamics**: Calculated upper-bound active-duration (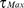) and optimal transcriptional burst size (*b^Opt^*) for each clone. Predicted large transcriptional bursts (*b^Opt^*≫1) identify clones for which the inferred transcriptional dynamics differ significantly from continuous transcription at a single fixed rate, and small 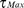 indicates good resolution of short bursts from less ‘noisy’ dynamics.

We find that the optimal agreement between model and experiment always occurs at short active-state durations (sample fits given in Fig. 3A), with deviations increasing for larger values of τ (Fig. 3B), past a distinguishability cut-off (where *Dev_r_* has increased by 1) that effectively marks the resolution limit of our analysis, which we call *τ_Max_* for each clone (Fig. 3C). The value of *τ_Max_* defines a range of active durations (bounded below by *τ* = 0), for which the quality of model fits for a given clone is comparable, and acts as a measure of how well our analysis can distinguish a ‘bursty’ underlying dynamic from other regime possibilities. Small values of *τ_Max_* indicate model fits where short-lived gene activation events, which are a hallmark of transcript production in bursts, provide a significantly better account of our experimentally measured distributions than a less noisy dynamic (i.e. one specified by longer active durations). Because we do find that the best model fits always occur at the shortest active durations (where the relative deviation *Dev_r_* = *Dev_r_^Opt^*, Fig. 3B), we conclude that a transcriptional dynamic in the ‘bursting’ regime does indeed always give the best *quantitative* account of our data, and we further note that larger predicted transcriptional bursts (generally associated with brighter clones) are correlated with better regime resolution (Fig. 3C). Finally, we note that our systematic distribution fitting procedure always resulted in improved fits over those obtained by only considering the first two distribution moments, with the improvement often statistically significant. Nevertheless, small systematic deviations remained, which are discussed further in Fig. S2.

### Transcriptional burst size is the primary feature that varies across viral integrations

**Figure 4:**
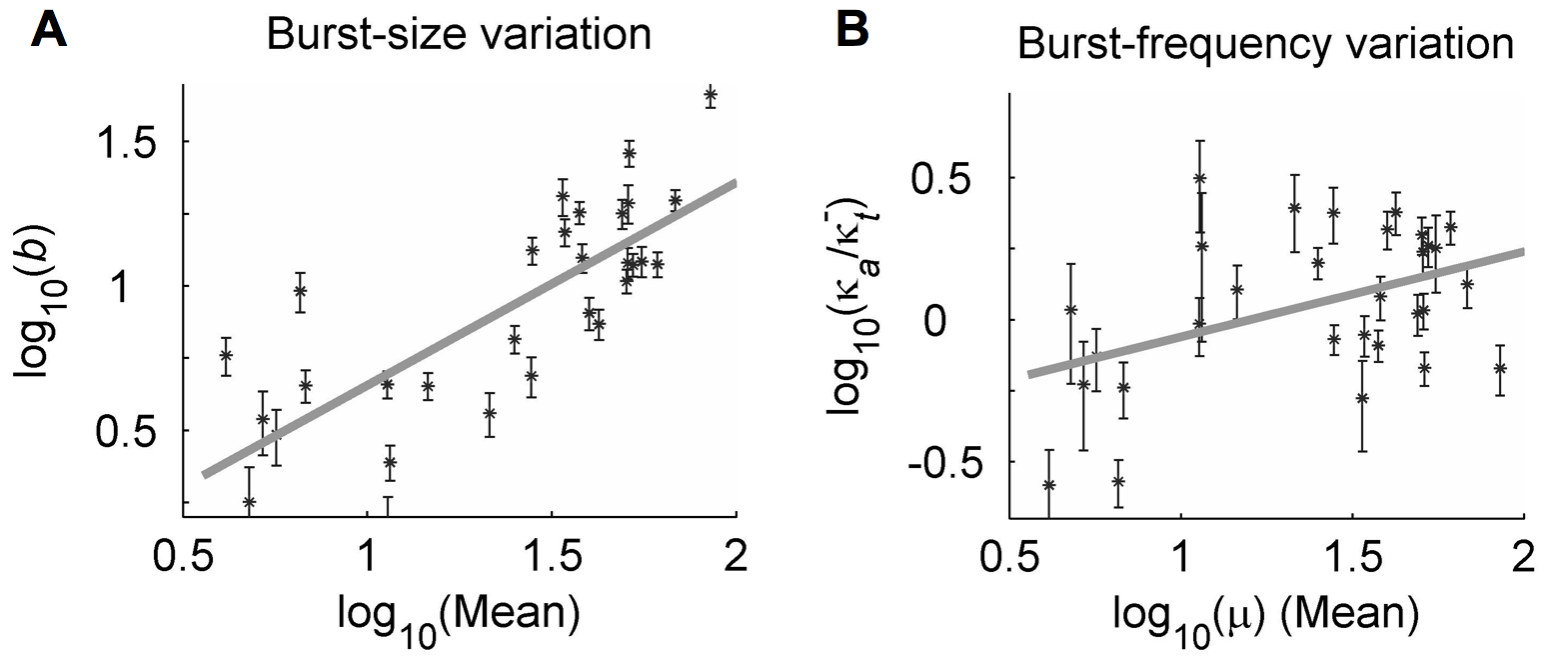
Modulation of transcriptional bursts by integration site. Best-fit transcriptional burst size (*b*) and burst frequency (*κ_a_*) that minimize the relative fit deviation (*Dev_r_*) were calculated at 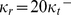 (which specifies a short active duration that was nearly optimal for all clones). Error bars represent the maximum 95% confidence interval for simultaneous parameter variations that increase *Dev_r_* by 1. Log-log regression coefficients represent power-law scaling of fit parameters with distribution mean (*μ*, measured in cytometer RFU), of the form 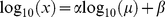 (*x* = *b* or *κ_a_*), and are given with 95% confidence intervals. **A**) *b*: α = 0.76±0.14; β = 0.13±0.2; *R^2^* = 0.66. **B**) *κ_a_*: α = 0.2±0.15; β = −0.5±0.2; *R^2^* = 0.2.

From the optimal fits above we identified best-fit transcriptional burst sizes and frequencies that specify the predicted transcriptional dynamics for each integration clone. Importantly, we find that the transcriptional burst size is the primary feature that varies over the set of genomic environments sampled by our 31 viral integrations, increasing from a few transcripts in very dim clones to tens of transcripts in very bright clones (Fig. 4A, with σ/μ = 3.5 for the distribution of log_10_(*b*)). Consistent with the qualitative analysis in Fig. 2C, we find that transcriptional burst size varies approximately sub-linearly with expression-distribution mean (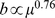, *R^2^* = 0.66). In contrast, the transcriptional burst frequencies inferred through our analysis are scattered about a characteristic value of one burst per several transcript degradation times, corresponding to several transcriptional bursts per cell-division time (Fig. 4B). In addition, these frequency values vary no more than several-fold (σ/μ = 2.2 for log_10_(*κ_a_*)), and they demonstrate little correlation with distribution mean (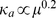, *R^2^* = 0.2). These results were maintained, to within the accuracy of our analysis, when the scattering gate used to control for cell-size variability in our experimental distributions was decreased by a factor of 6 from the value that was found to be optimal for our analysis (see Text S1, Sec. I and Fig. S4), indicating a robustness to this source of uncertainty, which had been found to significantly impact results from other cytometry-based analyses of expression variability [38]. Further, we find no significant correlation between the inferred transcriptional burst sizes and burst frequencies that might influence the interpretation of their trends with expression mean (see Fig. S5).

Our findings thus indicate that burst-size variation makes the dominant contribution in controlling single-gene expression profiles and represents the primary feature of transcriptional dynamics whose modulation distinguishes typical bright from dim clones. Importantly, the trends noted in Fig. 4 characterize the modulation of a ‘typical’ LTR integration by the sampled genomic environments. However, we must emphasize that the significant scatter of both the burst sizes and burst frequencies inferred for each individual clone about these ‘typical’ variations, as well as the possibility that a different trend may exist for very dim integrations (which were not considered in this study), suggest a potentially richer behavior that may still be uncovered through further study.

Another recent study has also considered a two state model to analyze expression variability from the HIV LTR [56]. This study similarly suggests that transcript production occurs in bursts and that both burst size and frequency vary with LTR integration position, though the analysis is qualitative, based only on consideration of distribution moments. In contrast to our findings, they emphasize burst frequency modulation as structuring distribution-shape variation over integration positions, as well as in response to pharmacological perturbation, though the later finding is difficult to interpret, as a steady-state model is considered to analyze data that are clearly varying in time. Additional quantitative analysis, including systematic model fitting, would be necessary to characterize the relative contributions of burst-size and burst-frequency modulation in this study, and to determine whether its findings are consistent with our own.

### Distinguishing modes of integration-site regulation of transcriptional dynamics

**Figure 5:**
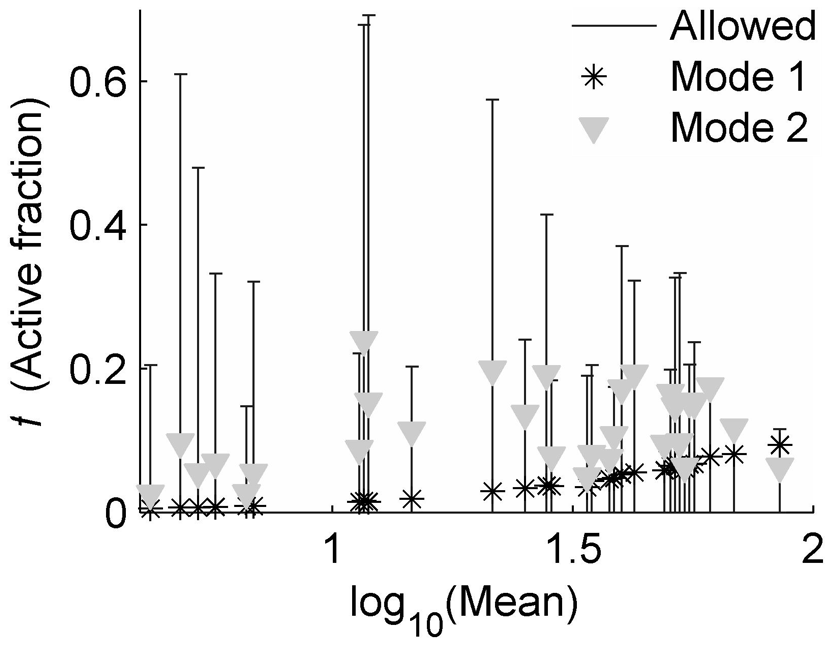
Active-fraction variation distinguishes modes of transcriptional regulation. The best-fit restriction of τ below τ*^Max^* in Fig. 3 specifies an upper bound on the predicted fraction of cells with the LTR in the active state (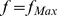), which is marked by bars for each clone. Each **Mode** of integration-site modulation of transcriptional dynamics leads to a different expected variation of 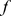 that distinguishes them. For **Mode 1**, active-state stability varies over integration clones, with the active-state transcription rate fixed (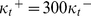 was used for this example), while for **Mode 2** the active-state transcription rate varies over integrations, with the active duration fixed (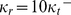 was used for this example).

A correlate of our findings – that transcription in short bursts underlies basal expression heterogeneities from the HIV LTR in the absence of Tat – is that the active promoter configuration is short-lived. This implies that the promoter would be observed in the active configuration for only a small fraction of cells in a clonal population at any given time at steady state. The value of this fraction in the two-state model, which we refer to as the ‘active fraction’, *f*, is related to the activation frequency (*κ_a_*, whose value is relatively well resolved for each clone by our analysis, Fig. 4B) and active duration (*τ*, for which our analysis only provides an upper bound *τ_Max_*, Fig. 3C), as 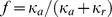 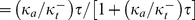. Our analysis provides a predicted upper bound on this fraction for each clone as 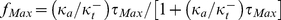 (Fig. 5, bars), where any value of *f* below 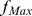 is consistent with our analysis. Small values of 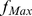 specify clones for which the active fraction is indeed predicted to be small, while larger values indicate clones for which its value is less well resolved. In particular, our analysis predicts that although the brightest and dimmest integration clones considered in our study differ in mean expression by a factor of approximately 30, the brightest clones will nevertheless only be observed with the integrated LTR in the ‘active’ transcript-producing configuration less than 20% of the time.

Transcriptional burst size – defined by the ratio of the transcription rate to promoter-inactivation rate (or the product of transcription rate and the active duration 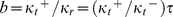) – can be modulated by two qualitatively difference ‘Modes’ of regulation. First, integration position could affect the dynamics of promoter inactivation (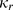), reflecting integration-position effects on the stability of the active configuration, possibly due to direct effects of the surrounding chromatin configurations on the energetics of the active configuration and/or the stabilizing effects of regulatory factor recruitment by surrounding regions (Mode 1). Alternately, integration position could affect transcriptional productivity in the active state (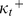), which may also be affected by modulation of chromatin configuration and/or recruitment of regulatory factors by surrounding genomic regions (Mode 2). We have seen in our analysis to this point, that model fits of our cytometry data cannot separate the two constituent parameters that define transcriptional burst size, and therefore they cannot resolve these two possible ‘Modes’ of its regulation (e.g. Fig. 3; a similar parametric indeterminacy has been noted by [46], [48]). Furthermore, the potentially overlapping effects of many molecular regulatory mechanisms on transcriptional dynamics may make it difficult to define experiments that directly distinguish these ‘Modes’, and to decouple their regulatory contributions.

However, our analysis predicts that each ‘Mode’ of control leads to a distinct pattern of active-fraction variation over the set of integration clones (Fig. 5, symbols): for Mode 1 the active-fraction varies proportionately to the clonal expression mean, whereas for Mode 2 the scatter in active fraction predicted over our set of integration clones reflects scatter in the predicted burst frequencies. We thus suggest that future experimental analysis of the active fraction may provide a means of distinguishing between these two key ‘Modes’ of integration-site modulation of gene expression.

## Discussion

Our findings, that expression from the HIV promoter is characterized by transcript production in bursts and that the site of viral integration primarily modulates transcriptional burst size, contribute to an emerging paradigm for transcriptional regulation that emphasizes the importance of stochastic/probabilistic dynamics [20], [27], [50], [57]. In particular, the expression patterns that we observe from single integrations of the HIV promoter cannot be accounted for by transcription from a single, fixed state of promoter activation, which would involve a single transcription rate that specifies a comparatively narrow single-gene expression profile with little variation over a population of cells. Rather, our analysis predicts that the large expression heterogeneities observed in this system (Fig. 1) are shaped by probabilistic transitions between (at least) two distinct configurations (Fig. 2A), with the promoter spending only the minority of time in the transcriptionally active configuration even for the most productive integrations (Fig. 2B, Figs. 3B,C and Fig. 5). Furthermore, our analysis suggests that an essential component of the regulatory effect of genomic environment at the viral integration site is to modulate the *dynamics* of transitions between states of differing transcriptional activity, in addition to possible effects on the transcriptional activity of each state (Figs. 4 and 5). Of note, it is only by systematically fitting a quantitative model to our measurements that these underlying dynamics were revealed, as quantitative single-cell measurements of protein expression only provide an indirect measure, and it is only by applying our systematic analysis to observations across a diverse sampling of integration-modulated expression patterns that we succeeded in extracting a characteristic effect of integration position on transcriptional dynamics.

### What features of the HIV-LTR determine ‘bursty’ transcription?

Transcript production in bursts is a particularly ‘noisy’ transcriptional dynamic that can generate significant cell-to-cell expression variability, which is reflected in wide and highly skewed single-gene expression distributions across clonal populations (Fig. 1C, 2B). In particular, the ‘typical’ distribution identified in Fig. 1C demonstrates a coefficient of variation (standard deviation/mean, or relative width), corresponding to 60% variability. This value is significantly larger than the values observed for most eukaryotic promoters in several large-scale studies (compare to data in: [38], [39], [58]), and we anticipate that a number of features of the HIV promoter, some of which are common in mammalian promoters, may conspire to account for this ‘noisy’ expression pattern.

Similar to HIV expression shortly after infection, our system lacks the viral transcriptional activator Tat. In the absence of Tat the LTR has been observed to bind repressive factors that maintain a non-conducive chromatin configuration [14], , and the likely greater stability of this ‘inactive’ configuration may limit the fraction of time that a transcriptionally ‘active’ configuration can be maintained. On the other hand, like many mammalian promoters, the HIV LTR contains multiple binding sites for repressing and activating elements (which still bind in the absence of Tat), several of which affect chromatin state and bind competitively and/or cooperatively. For example, the histone-acetyltransferase (HAT) p300 and the activating NF-κB component RelA are thought to bind their respective HIV-1 Sp1 and NF-κB sites cooperatively to activate transcription, and in competition with the histone deacetylase (HDAC) recruiting activity of SP1 and the p50/p50 homo-dimer that bind the same sites respectively to inhibit transcription [10], [62]–[66]. One may hypothesize that this competition could thus lead to an infrequent all-or-none binding of activating factors that directly remodel promoter-bound nucleosomes to establish a transcriptionally conducive chromatin configuration [10]. In addition, the LTR includes a number of other cis-regulatory elements that bind transcriptional activators such as NFAT and AP-1 [67], [68], as well a TATA motif that contributes core transcriptional complexes [69], [70]. These elements may enable more efficient recruitment, assembly, and stabilization of a productive transcription complex, with transcriptional reinitiation potentially yielding multiple transcripts from each gene-activation event (the presence of a TATA box has been linked to increased expression noise in other studies as well, see for example: [41], [47], [71]). In combination, the above features may specify transcript production during short, infrequent bursts, consistent with the results of our analysis.

Intriguingly, a recent mammalian genome-wide mapping of HAT and HDAC association found them simultaneously bound to a large number of active promoters, suggesting that simultaneous regulation by competitive epigenetic regulators may be more common than previously thought [72]. It is therefore possible that transcript production in bursts represents a more general feature of mammalian expression regulation, and it will be interesting to discover how properties of the HIV promoter shape its transcriptional dynamics, and whether similar promoter architectures specify ‘bursty’ dynamics for other genes.

### Significance of burst-size variation over LTR genomic integrations

Our findings suggest that transcriptional burst size is a more ‘locally’ determined property, more sensitive to those features of genomic environment that vary significantly between integration sites, whereas transcriptional burst frequency is, by comparison, a more ‘globally’ determined feature, specified by interactions with the cellular environment that may be more promoter-specific but less significantly integration-site dependent. Burst frequency reflects the statistics of assembling the more active promoter configuration from an inactive one, and we might speculate that this transition depends in part on large-scale chromatin reorganization and dynamics that are coordinated globally across the nucleus [73], [74], [75]. Burst size, on the other hand, is a property of the transcriptionally ‘active’ configuration, and we may conjecture that some of the reorganization that accompanies its establishment also may provide opportunities for important ‘local’ features to exert their regulatory influences. For example, chromatin remodeling may expose new binding sites for transcriptional regulators [31], [76], and the initiation of transcriptional activity could contribute to association with ‘nearby’ transcription factories where additional transcriptional regulators are localized, and where interactions with surrounding (and possibly distant) genomic regions may be enhanced [73].

At a more basic level, a feature of transcriptional burst size that could more generally account for a greater sensitivity to genomic environment is its complimentary dependence on transcriptional productivity and the stability of the active promoter configuration. We had noted earlier that this complimentary dependence specifies two distinct ‘Modes’ by which surrounding genomic regions may differentially affect the resulting transcriptional dynamics (see Fig. 5), both of which might be effected by recruitment of transcriptional regulators by surrounding genomic regions, epigenetic features of the surrounding regions, and the transcriptional activity of neighboring genes [15], [75], [77]. If we assume that a ‘typical’ more productive integration increases 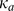, τ, and 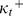 all proportionately (i.e. without assuming a weaker dependence for burst frequency), then the dual dependence of burst size would dictate that it vary as burst frequency squared (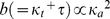), and the scalings 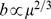 and 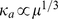 would result, which fall within the 95% confidence interval of our regression analysis in Fig. 4. This possibility is consistent with our suggestion in the previous subsection that the architecture of the HIV LTR may effectively couple the control of gene activation and inactivation, and with the hypothesis that the chromatin regulators that may control these dynamics could also modulate the active-state transcription rate either directly or indirectly. Such a combined ‘Mode’ of modulation would specify an active-fraction variation intermediate between that predicted for the two pure ‘Modes’ of modulation considered in Fig. 5, and might be used to distinguish it experimentally.

Burst-size variation with promoter induction level from a tetracycline-inducible construct at a single genomic position has been noted in another study using mammalian cells [46], though this result contrasts with a number of yeast studies that have identified the frequency of gene-activation events as the primary feature that varies with genetic-construct induction level [45], [47], over a single set of three targeted genomic loci [36], and over a large set of endogenous promoters [38], [39]. It thus remains to be determined whether our observation of burst-size variation represents a mode of transcriptional regulation particular to mammalian cells or to transcriptional control by genomic environment, or whether it is determined by any specific features of the HIV promoter that dictate a unique coupling to mammalian genomic environments that might be shared by other ‘bursty’ promoters and cell types. Future studies investigating greater numbers of genomic integrations, in our and other systems, that correlate expression variability with promoter and surrounding genomic sequences, may provide important answers to such questions.

### Basal transcription as a determinant of HIV-infected cell fate

The observation that integration site primarily modulates transcriptional burst size from the HIV promoter implies that viral integrants sample a ‘noisy’ set of basal expression distributions by semi-randomly integrating in the genome. Specifically, relative distribution widths (i.e. the coefficient of variation) are approximately maintained and comparable between ‘dim’ and ‘bright’ integrations. This contrasts with the naive expectation that dimmer integrations should demonstrate greater relative expression heterogeneity due to larger relative fluctuations typically generated by smaller numbers of molecules, as would be the case if burst frequency were the primary covariate over viral integrations (see Fig. 2C), and as was found to be the case over a large sampling of yeast promoters [38], [39].

The basal expression patterns, and their associated expression noise, that were measured here reflect the range of expression dynamics that may be generated initially from an HIV infection after its semi-random integration into the host genome but prior to significant production of viral proteins [4], [9]. Productive viral replication depends on subsequent production of the HIV protein Tat, which mediates expression transactivation by enhancing both transcript elongation from the LTR as well as the binding of other transcriptional activators [51], [78]–[81]. In an intact virus, this positive feedback would act to amplify the basal expression fluctuations observed here.

**Figure 6:**
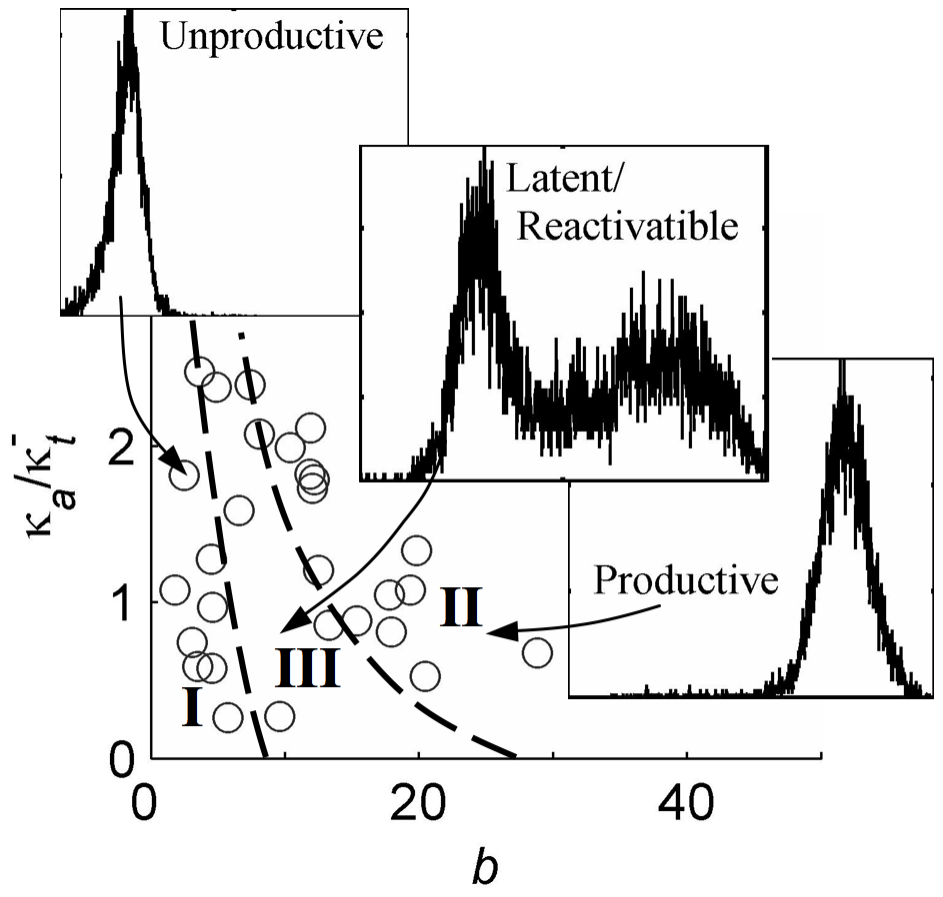
Basal promoter fluctuations as determinants of infected-cell fate. Possible decomposition of the ‘space’ of basal burst-parameters inferred by the current analysis into ranges of parameter combinations that, in the presence of positive feedback from Tat, may lead to active viral replication vs. latent fates. **Region I**: Basal transcription pattern never leads to Tat-transactivation. **Region II**: Fast transactivation always leads to a stable highly expressing state. **Region II**I: Bimodal expression patterns, where large fluctuations in basal transcriptional bursting can infrequently drive transitions from basal to transactivated states. Inset histograms demonstrate representative expression patterns for single-integration clones of a similar vector that includes Tat [4], [10], and region boundaries are hypothetical.

We anticipate that certain ranges of parameters representing integration-site dependent basal fluctuations in promoter activity may act to specify distinct infected-cell fates, as illustrated in Figure 6 where the drawn region boundaries are hypothetical and the insets depict representative expression phenotypes that result when Tat is expressed from the HIV LTR in a minimal viral system that we had studied in earlier work [4], [10]. Promoter integrations with smaller basal transcriptional burst sizes, and with frequencies that do not effectively couple one burst to the next, will never produce sufficient Tat for transactivation and may represent unproductive infections (**Region I**). On the other hand, promoter integrations specifying larger basal burst sizes and sufficient frequencies will quickly and stably transactivate after a small number of initial transcriptional bursts and may represent a productive infection (**Region II**). In contrast, those integrations with small to intermediate basal burst sizes and frequencies will only infrequently (stochastically) generate sufficient Tat for positive feedback activation. Moreover, the transactivated state may be subsequently destabilized by the infrequent occurrence of consecutive smaller and more widely spaced bursts, to generate a bimodal expression pattern (**Region III**). We have hypothesized that the dynamics of this phenotype, which include significant delays in switching between non-productive and productive expression phenotypes, may create a sufficient time window for the establishment of latent infections in vivo [4], [10]. Future experimental and computational analysis may provide additional insights into the role of Tat in amplifying basal, integration-modulated, expression fluctuations, as well as their hypothesized role in fate determination of HIV-infected cells.

### Implications for investigations of genomic architectures in health and disease

While other studies have considered the effects of genomic environment on mean expression, we have analyzed its effects on expression heterogeneities. By applying an integrated computational and experimental approach, we have characterized the modulation of underlying transcriptional dynamics by genomic environment in human cells. Since classes of human promoters often share common enhancer and repressor motifs, it is possible that two such promoters at different genomic loci would demonstrate significantly different transcriptional dynamics, as we have observed from different integrations of a single promoter in our system. In this way, genomic architecture would provide an additional axis of expression regulation complementary to that specified by individual promoter sequence architectures, and promoter and genomic architectures might evolve in parallel to optimize their coupled contributions to transcriptional control [72], [82]–[85]. Similarly, integrating viruses such as HIV, whose host-cell specificity determines the range of possible genomic environments that could be selectively sampled, may evolve promoter architectures that best exploit this host-regulatory axis to adopt a set of expression patterns that enhance, or even optimize, viral replication fate.

## Materials and Methods

### Harvesting and infection of lentivirus

The HIV-1 based lentiviral plasmid, pCLG, (encoding the HIV-1 LTR and GFP) was packaged and harvested in HEK 293T cells using 10 mg of vector, 5 mg pMDLg/pRRE, 3.5 mg pVSV-G, and 1.5 mg pRSV-Rev, as previously detailed [4], [86]. Harvested lentivirus was concentrated by ultracentrifugation to yield between 10^7^ and 10^8^ infectious units/ml. Approximately 10^3^–10^6^ infectious units of concentrated virus were used to infect 3×10^5^ Jurkat cells. Six days after infection, gene expression of infected cells was transactivated by incubating Jurkats with a combination of 20 ng/ml TNFα, 400 nM TSA, and 12.5 mg exogenous Tat protein [10]. After stimulation for 18 hours, GFP expression was measured by flow cytometry, and titering curves were constructed by determining the percentages of cells that exhibited GFP fluorescence greater than background levels. This titering curve was used to attain the desired MOI (∼0.05–0.10).

### Clone selection and FACS analysis

Forty-eight single GFP+ LTR-GFP (LG) Jurkats (clones) were sorted on a DAKO-Cytomation MoFlo Sorter into 96-well plates and cultured for at least 4 weeks to allow for clonal expansion. Infected cultures were analyzed via flow cytometry on a Beckman-Coulter EPICS XL-MCL cytometer (http://biology.berkeley.edu/crl/cell_sorters_analysers.html). Thirty-one single-integration clones, whose expression histograms were sufficiently distinguishable from an autofluorescence profile for model fitting (with mean fluorescence exceeding twice the autofluorescence mean), were selected for further analysis.

### Distribution processing

Cytometry measurements on 10^4^ cells for each clone quantified GFP fluorescence as well as forward and side scatter (FSC and SSC). Live cells were selected by standard gating of FSC and SSC, and further gated to select the mid 60% of FSC and SSC values. This gating was optimized using a bootstrap approach to resolve the GFP profile at the mean scattering measure, while eliminating significant correlation between GFP distribution and scattering (see Text S1 Sec. S.I for further discussion, Fig. S1, and Table S1). GFP histograms were smoothed using an optimized low-pass Fourier filter, and normalized to obtain probability distributions, that were used for model fitting. Distribution deconvolution, for the transformation applied in Fig. 2B, was accomplished using a Weiner filter. Model fits were also obtained for distributions resulting from a 10% scattering gate, and indicate no significant effect on our parameter inferences (Fig. S4).

### Model solution

The model in Fig. 2A represents a continuous-time discrete-state Markov process described by a chemical master equation [87], which was solved using an in-house Matlab routine (The MathWorks, Inc.; code available upon request) for steady-state protein distributions, which were then convolved with a separately measured autoflorescence profile and converted to cytometer-based RFU (Relative Fluorescence Units) values for comparison with smoothed experimental distributions. Briefly, the master equation was truncated at large protein and transcript numbers to specify a finite system. A graded coarse-graining approach was applied, whereby neighboring states at higher transcript and/or protein number, where distributions admit a continuum approximation, were binned together (probabilities summed), and transition rates between binned states were approximated by interpolation to estimate probability fluxes at the boundaries. The coarse graining scheme reproduces the master equation at small transcript and protein numbers (where no coarse-graining is applied), and specifies a second-order approximation to the corresponding Fokker-Planck equation at large protein and transcript numbers. The resulting linear system was then integrated in time until an effectively stationary distribution was achieved by using a forward/backwards Euler method that alternates treating transcript and protein transitions implicitly or explicitly; this represents a fast and stable method, appropriate to stiff systems and multi-dimensional PDEs [88]. Marginal coarse-grained protein distributions were then calculated by summing calculated probabilities over transcript numbers and gene states, and the resulting distributions were interpolated. Solution accuracy was established by comparing the first three moments of the calculated distributions to their theoretical values (calculated analytically, see Text S1, Sec. S.III), by varying the coarse graining and the time step for the integrator, and by comparing our solutions to those calculated using the Finite State Projection algorithm developed by Munsky and Khummash [89], which allows a rigorous calculation of numerical error for finite times, for several test cases. Further details may be found in Text S1 Sec. S.II.

### Fitting procedure

Fit parameters (*κ_a_*, *b* = *κ_t_^+^*/*κ_r_*, and *κ_r_*) were varied using the MATLAB minimization function ‘fmincon’ to identify the combination that minimized the fit deviation, defined as 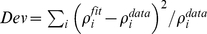, where 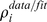 is the predicted/measured probability of counting a cell in cytometer bin *i* for the *data/fit*.

### Specifying non-fit model parameters

A number of model parameters quantify processes occurring at spatially separate locations from the integrated LTR. These were assumed to be the same for all integrations, and were specified separately. Methods developed independently from this study allowed us to calibrate relevant non-fit model parameters via comparison between the measured transcript distribution for a single clone, and the corresponding cytometry-based GFP distributions (Foley, et al. manuscript in preparation). A conversion factor between transcript number and RFU could be estimated from the measured ratio of means, as 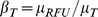 = 2.5 (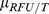 = measured cytometry-based RFU/transcript mean). By assuming transcriptional bursting, the ratio of transcript to protein degradation rates could be calculated as 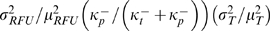, yielding a value of 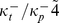 for our measurement. These constitute the remaining quantities necessary to specify our model. While uncertainties in these quantities would affect the values inferred for model fit parameters, they would approximately affect the inferred fit parameters for each clone by the same scale factor, preserving inferred trends in parameter variation over the set of integration clones. These uncertainties were therefore not explicitly considered in our analysis (see Text S.1, Sec. S.VII for further discussion). Quantifying the dilution of a synthetic non-degraded fluorescence marker allowed us to estimate a cell-division rate of 0.05 h^−1^, which served as an effective protein degradation rate (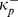) in our model, and thus specified 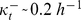; the absolute values of these degradation rates were not essential to specifying our model because steady-state distributions only depend on ratios of rate constants, and all rates were therefore scaled relative to the transcript degradation rate in our analysis. The relatively large protein numbers in our system dictate that fluctuations in protein production and degradation do not significantly influence distribution shapes, and as long as the ratio 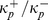 was chosen to be a sufficiently large value, its specific value did not affect our analysis; we chose 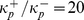. See Text S1, Sec. S.V for further discussion of non-fit model parameters.

### Quantifying experimental uncertainties and model-fit discrimination

A bootstrap procedure was used to estimate a 95% upper-bound on the value of *Dev* for our processed experimental distributions (*Dev_data_*) that included uncertainties due to the finite number of cells sampled and to specifying distributions at a single scattering measure. Other sources of uncertainty, such as cytometer PSF and distribution variability over time, were found not to significantly affect our determination of trends in model-parameter variations over the set of integration clones and were not included (see Text S1, Sec. S.VIII, and Fig. S3). Model fits whose deviations (*Dev_fit_*) differed from each other by less than *Dev_data_* were considered effectively indistinguishable, as the differences in their quantified deviations might be accounted for by uncertainty in our experimental distributions. 95% confidence intervals about best-fit model parameters were calculated as maximum variations for which the increase in *Dev_r_* = *Dev_fit_*/*Dev_data_* was less than 1 (assuming simultaneous parameter variations), as estimated using a Hessian-based quadratic approximation for variation of *Dev_r_* with respect to burst parameters and based on the parametric sampling in Fig. 3 for *κ_r_*.

## Supporting Information

Figure S1Distribution processing. A) 2-d histogram of fluorescence and forward scatter (FSC) values, as measured by cytometry from 10^4^ cells, for a sample clone. FSC is binned on a linear axis covering values between 1 and 1024 (10 bits) in arbitrary units (AU), and fluorescence values were log-binned over 4 orders of magnitude in relative florescence units (RFU). B) Smooth 2-d histograms were generated using a low-pass Fourier filter. The dashed line highlights correlation between fluorescence and FSC measures (we aim to account for this correlation in a distribution-processing procedure), and the green line is drawn at the mean FSC value, which specifies C) the ‘target’ GFP distribution at fixed FSC that we aim to extract by our processing procedure. D) Optimized gating. For each clone, a bootstrap approach was used to determine the optimal fraction of the FSC range to gate the data by (% Gate), which for each clone, minimizes the average over the set of re-sampled (synthetic) data of the deviation between each processed ‘synthetic’ data set and the ‘target’ distribution. The distribution deviation is defined as in the main text, as 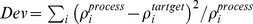, where 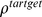 is the target distribution, 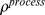 is a processed synthetic distribution, both have been normalized as probabilities, and the sum is taken over cytometry bins. 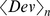 marks the calculated value of *Dev* for each clone, averaged over the set of synthetic data, and normalized by the optimal value. The box plot shows the dependence of 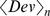 on the % Gate over the full set of clones that were fit, with box edges marking the inter-quartile range (iqr), whiskers marking 1.5*iqr ( = 2σ for a normal distribution), and ‘+’ marking outlier clones. Though the minimal (optimal) value of 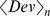 often occurs for a gate slightly narrower than 60%, 60% is nearly optimal for all clones and was used to process our data for analysis and fitting. E) Alternate corrections. The average value of 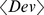 over the set of clones (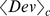), for each gate size, is calculated for the different corrections mentioned in the text. Subtracting linear correlation (‘*lin*’), or dividing the GFP by FSC values (‘*Div*’), makes little difference compared to making no further correction (‘*Cor*’) in reproducing the ‘target’ distribution. F) Fractional contribution of cell-size variability to expression heterogeneity. For each clone, after applying a 60% gate to select cells in the middle of the FSC range, the fraction of the variance of the GFP histogram that can be attributed to FSC variation (i.e. cell size variability), is calculated as the *R* ^2^ value for a linear regression between FSC and GFP, equal to 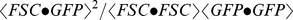, where the brackets denote the population average. G, H) Gating for cell size has little effect on distribution noise and skew. Square gates in the FSC/SSC plane were defined by taking varying percentages of the total cells for each clonal population about the mean FSC and SSC values (Gate %). For each Gate %, the distribution coefficient of variation (CV = σ/μ, G), or skewness (‘skew’ = *m_3_*/σ^3^, *m_3_* = 3^rd^ distribution central moment, H), was calculated for each clone, relative to the value measured at the 60% gate used in the main text. The mean value of this ratio over the set of integration clones is given by the red line at each Gate %, with the box marking the inter-quartile range (iqr) and the bar marking 1.5*iqr.(1.09 MB TIF)

Figure S2 Fit quality and deviations. A) Fit uncertainty. The relative fit deviation (*Dev_r_*) for each clone, defined as the ratio of *Dev* for the fit to a 95% upper-bound on the value expected due to uncertainty in our experimental distribution (see main text and Sec. S.I), are plotted for the systematic fits described in the main text (‘Sys’) and for the moment fits (‘Mom’) described in Sec. S.VI. Values of *Dev* below 1 (marked by the dashed line) indicate fits that cannot be significantly improved, within the resolution of our data. B) Fit improvement. The decrease in *Dev_r_*, in going from the initial moment-based fits to systematic fits (δ*Dev_r_*), is given, with values greater than 1 (marked by the dashed line) indicating clones whose fits were significantly improved by the systematic fitting procedure. C) Shape of the fit deviation. For each of our 31 distribution fits, the deviation between the model prediction and the smooth experimental data is plotted on the log-binned fluorescence axis on which our cytometry data was ginned. Each fit deviation is scaled so that its peak absolute value is equal to 1, and each was translated to a mean of 100 RFU to superimpose them. Deviations were only calculated for bins whose probability was greater than 2% of the distribution maximum. D) Fit-deviation significance. The data in C is re-plotted with the deviation at each bin normalized by the bootstrap estimated 95% upper bound on its expected value due to uncertainty in our data, as calculated in Sec. S.I. Absolute values greater than 1 (marked by the dashed lines) indicate bins for which the fit deviation is significant.
(1.87 MB TIF)

Figure S3 Distribution stability over time. A) Distribution variation over time is not correlated among clones. Six clones and a control with no plasmid that quantifies cellular autofluorescence (‘Aut’) were followed over 6 consecutive days by cytometry. Daily fluctuations in fluorescence mean (μ_t_, normalized by the value on the first day μ_0_, for each clone) are uncorrelated over the sampled populations for any pair of time points (P>0.5). B) Distribution shape variations over time for any clone are approximated by a distribution scaling of all fluorescence by a constant value, such that the variance (σ^2^) changes approximately as the mean squared (μ^2^). For small deviations, this translates as the relative variance (σ_t_^2^ at each time, normalized by its value on the first day, σ_0_^2^ for each clone) changing in proportion to twice the change in mean, which is plotted as a reference line (‘Scaling’). C) Distribution variability over time for a sample clone approximately demonstrates a ‘scaling’ variation, as noted in B, which is equivalent to translating the distribution on the log-binned fluorescence axis on which the histogram is plotted. D) Distribution rescaling. For the sample clone in C, the fluorescence values each day are scaled by the ratio of the mean on the first day to the mean on that day. This rescaling leads to improved distribution stability over time. In particular, the distribution variability is now approximately within the experimental uncertainty due to our distribution-processing procedure. This suggests that distribution drift over time can be treated as a simple scaling of fluorescence values, perhaps due to metabolic drift, as discussed in the text. E, F) Best-fit model parameter variability over time is comparable to 95% confidence intervals calculated for sources of uncertainty considered in the main text. For each clone, the fitting procedure of the main text was applied to each processed experimental distribution, for each of the six days. Best-fit transcriptional burst frequencies (E) and burst sizes (F) for each clone, relative the value obtained for fitting the average of its distribution over time, is plotted against the log expression mean (averaged over the six days). Bars about the value of 1 represent 95% confidence intervals, as calculated in the main text, which do not include uncertainty due to distribution variability over time.
(0.86 MB TIF)

Figure S4 Gating for cell size does not significantly affect inferred trends in burst-parameter variation with integration position. A, B) The experimental distributions obtained by applying a 10% square gate in the FSC/SSC plane (as discussed in Sec. S.I.7) were fit following the procedure in the main text (‘narrow gate’), and the resulting best-fit model parameters compared to those obtained for each clone based on our optimized distribution processing procedure (‘optimal gate’,  = 60%), that were given in Fig. 4. Bars represent 95% confidence intervals, as obtained in the main text. Fit parameters for the ‘narrow gate’ data only demonstrate slight differences from the ‘optimal gate’ data, and demonstrate no significant difference in trend with expression mean, confirming that our results are robust to gating for cell size.
(0.55 MB TIF)

Figure S5 No significant correlation between transcriptional burst size and frequency for the HIV LTR. The best-fit transcriptional burst frequencies (*κ_a_*), which were inferred for our system in Fig. 4 of the main text, are plotted against the corresponding inferred transcriptional burst size (*b*) for each clone (*) in a log-log plot to investigate possible correlations. Diagonal lines (green) represent combinations of burst size and frequency that specify constant mean expression (μ ∝ *κ_a_ b* in the bursting regime). The 95% confidence region calculated in the main text are ovals in the *b*×*κ_a_* plane, and become deformed to rounded crescents in the log-log plot, represented by the closed curves about each combination of best-fit parameters (blue). Notice that in the region closest to the each best-fit parameter combination, the corresponding confidence boundary generally depicts less constrained variation in the direction that preserves expression mean (i.e. parallel to the drawn lines of constant mean). Linear regression reveals a slope of 0.17±0.3 (95% confidence), with an *R^2^* value of 0.05, and a Pearson correlation coefficient of 0.2. We thus conclude that significant correlations between burst size and frequency, that might affect the trends in these parameters with distribution mean that were analyzed in Fig. 4 of the main text, are not present in our system.
(0.35 MB TIF)

Table S1 Work flow.
(0.97 MB TIF)

Text S1 Supplement to “HIV-Promoter Integration Site Primarily Modulates Transcriptional Burst Size, Rather Than Frequency.”
(0.89 MB PDF)
