# Supplementary figures and images for: HIV Promoter Integration Site Primarily Modulates Transcriptional Burst Size Rather Than Frequency
Source: PLoS Comput Biol. 2010 Sep 30;6(9):e1000952. doi: 10.1371/journal.pcbi.1000952 (PMC2947985; doi:10.1371/journal.pcbi.1000952)

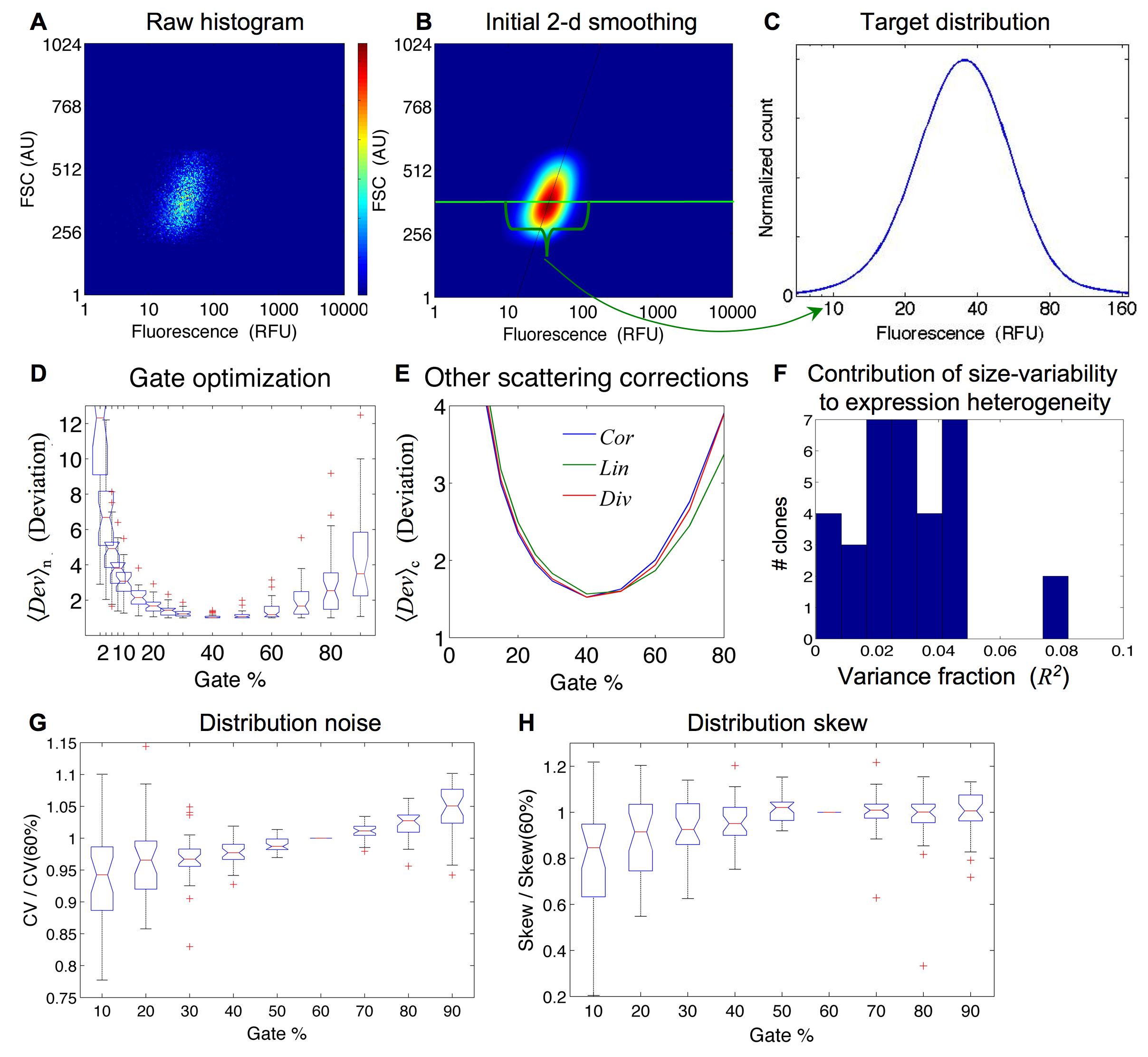

Supplement: Figure S1 — Distribution processing. A) 2-d histogram of fluorescence and forward scatter (FSC) values, as measured by cytometry from 104 cells, for a sample clone. FSC is binned on a linear axis covering values between 1 and 1024 (10 bits) in arbitrary units (AU), and fluorescence values were log-binned over 4 orders of magnitude in relative florescence units (RFU). B) Smooth 2-d histograms were generated using a low-pass Fourier filter. The dashed line highlights correlation between fluorescence and FSC measures (we aim to account for this correlation in a distribution-processing procedure), and the green line is drawn at the mean FSC value, which specifies C) the ‘target’ GFP distribution at fixed FSC that we aim to extract by our processing procedure. D) Optimized gating. For each clone, a bootstrap approach was used to determine the optimal fraction of the FSC range to gate the data by (% Gate), which for each clone, minimizes the average over the set of re-sampled (synthetic) data of the deviation between each processed ‘synthetic’ data set and the ‘target’ distribution. The distribution deviation is defined as in the main text, as , where is the target distribution, is a processed synthetic distribution, both have been normalized as probabilities, and the sum is taken over cytometry bins. marks the calculated value of Dev for each clone, averaged over the set of synthetic data, and normalized by the optimal value. The box plot shows the dependence of on the % Gate over the full set of clones that were fit, with box edges marking the inter-quartile range (iqr), whiskers marking 1.5*iqr ( = 2σ for a normal distribution), and ‘+’ marking outlier clones. Though the minimal (optimal) value of often occurs for a gate slightly narrower than 60%, 60% is nearly optimal for all clones and was used to process our data for analysis and fitting. E) Alternate corrections. The average value of over the set of clones (), for each gate size, is calculated for the different corrections me [file pcbi.1000952.s001.tif]

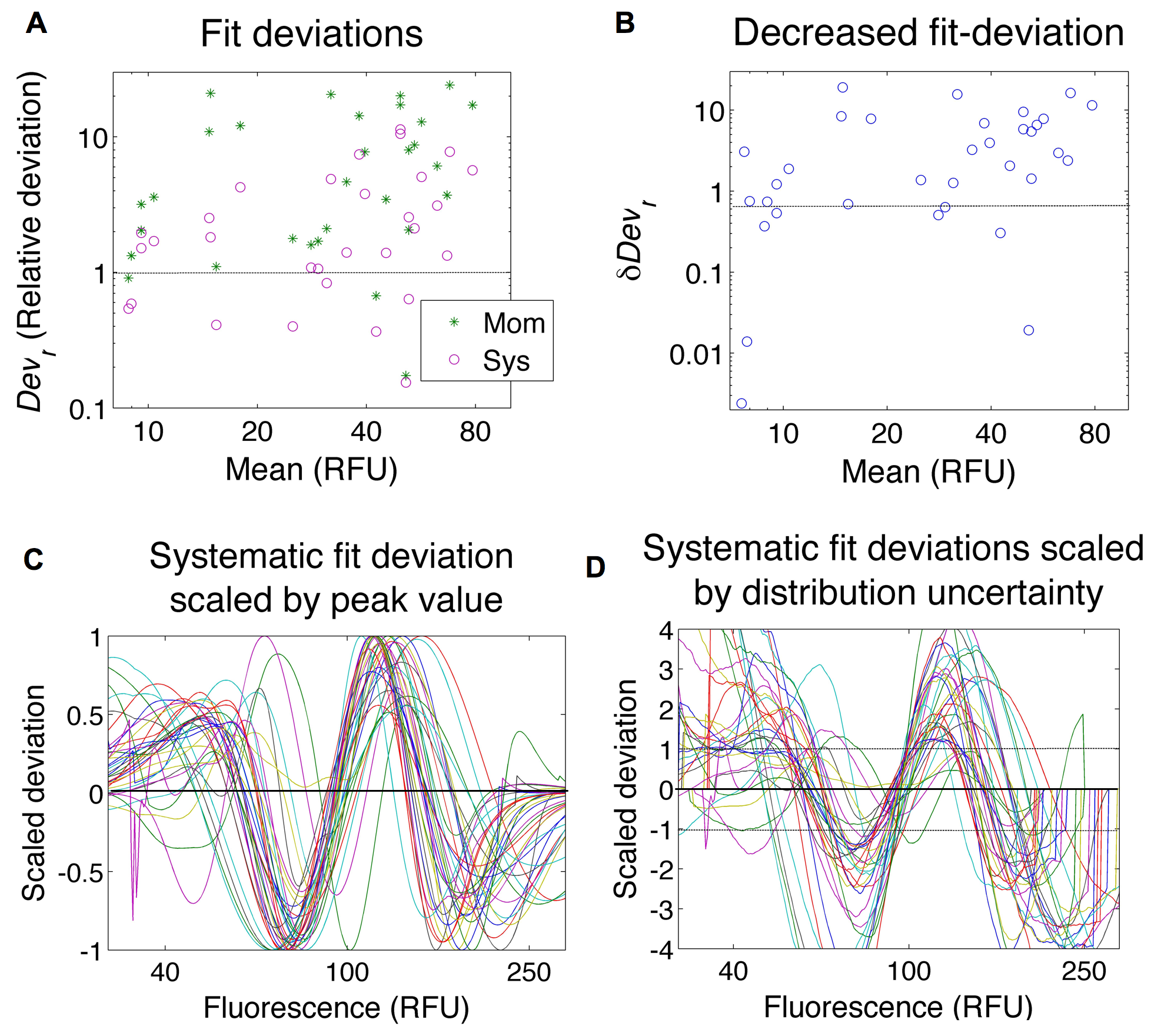

Supplement: Figure S2 — Fit quality and deviations. A) Fit uncertainty. The relative fit deviation (Devr) for each clone, defined as the ratio of Dev for the fit to a 95% upper-bound on the value expected due to uncertainty in our experimental distribution (see main text and Sec. S.I), are plotted for the systematic fits described in the main text (‘Sys’) and for the moment fits (‘Mom’) described in Sec. S.VI. Values of Dev below 1 (marked by the dashed line) indicate fits that cannot be significantly improved, within the resolution of our data. B) Fit improvement. The decrease in Devr, in going from the initial moment-based fits to systematic fits (δDevr), is given, with values greater than 1 (marked by the dashed line) indicating clones whose fits were significantly improved by the systematic fitting procedure. C) Shape of the fit deviation. For each of our 31 distribution fits, the deviation between the model prediction and the smooth experimental data is plotted on the log-binned fluorescence axis on which our cytometry data was ginned. Each fit deviation is scaled so that its peak absolute value is equal to 1, and each was translated to a mean of 100 RFU to superimpose them. Deviations were only calculated for bins whose probability was greater than 2% of the distribution maximum. D) Fit-deviation significance. The data in C is re-plotted with the deviation at each bin normalized by the bootstrap estimated 95% upper bound on its expected value due to uncertainty in our data, as calculated in Sec. S.I. Absolute values greater than 1 (marked by the dashed lines) indicate bins for which the fit deviation is significant. (1.87 MB TIF) [file pcbi.1000952.s002.tif]

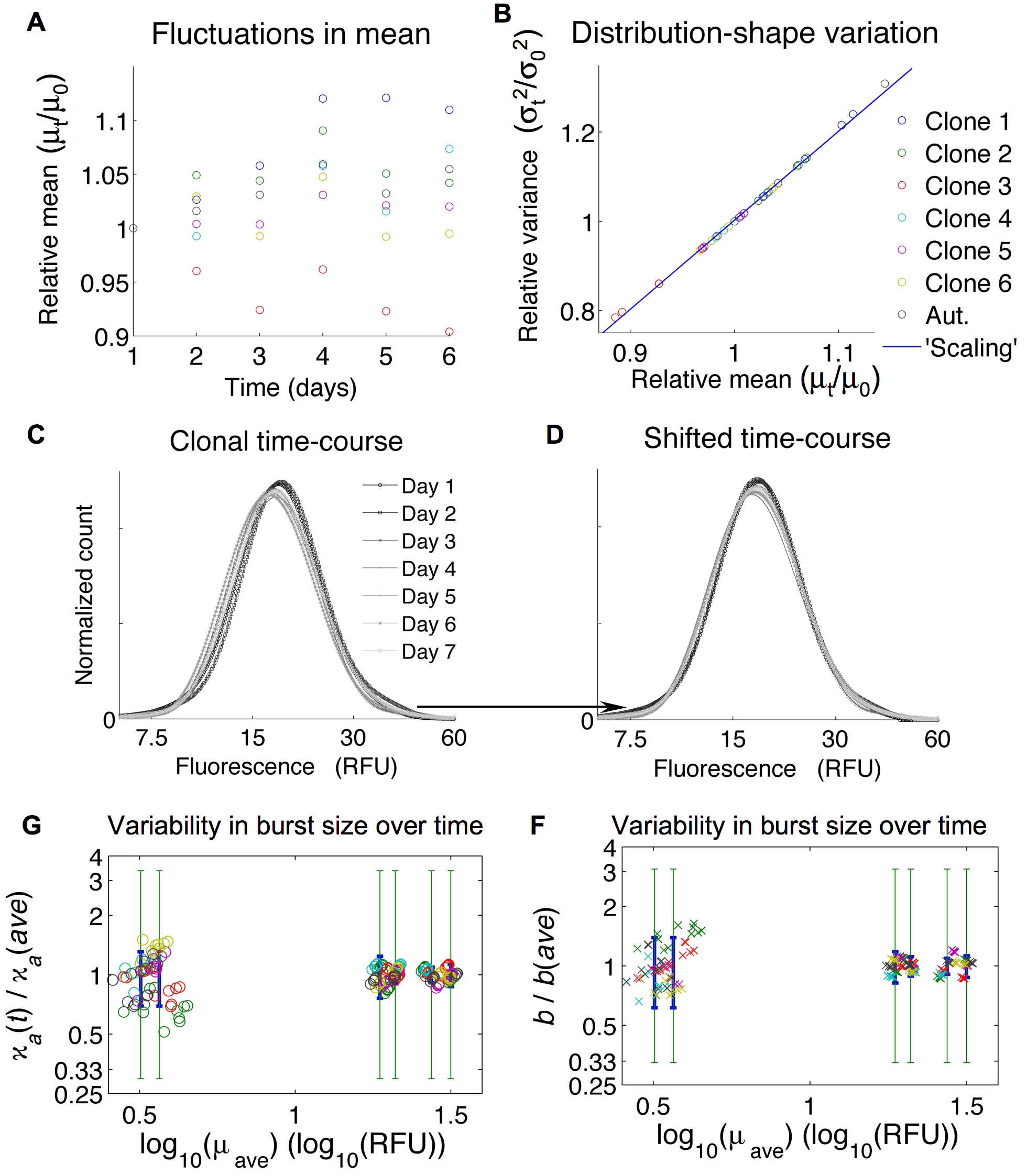

Supplement: Figure S3 — Distribution stability over time. A) Distribution variation over time is not correlated among clones. Six clones and a control with no plasmid that quantifies cellular autofluorescence (‘Aut’) were followed over 6 consecutive days by cytometry. Daily fluctuations in fluorescence mean (μt, normalized by the value on the first day μ0, for each clone) are uncorrelated over the sampled populations for any pair of time points (P>0.5). B) Distribution shape variations over time for any clone are approximated by a distribution scaling of all fluorescence by a constant value, such that the variance (σ2) changes approximately as the mean squared (μ2). For small deviations, this translates as the relative variance (σt 2 at each time, normalized by its value on the first day, σ0 2 for each clone) changing in proportion to twice the change in mean, which is plotted as a reference line (‘Scaling’). C) Distribution variability over time for a sample clone approximately demonstrates a ‘scaling’ variation, as noted in B, which is equivalent to translating the distribution on the log-binned fluorescence axis on which the histogram is plotted. D) Distribution rescaling. For the sample clone in C, the fluorescence values each day are scaled by the ratio of the mean on the first day to the mean on that day. This rescaling leads to improved distribution stability over time. In particular, the distribution variability is now approximately within the experimental uncertainty due to our distribution-processing procedure. This suggests that distribution drift over time can be treated as a simple scaling of fluorescence values, perhaps due to metabolic drift, as discussed in the text. E, F) Best-fit model parameter variability over time is comparable to 95% confidence intervals calculated for sources of uncertainty considered in the main text. For each clone, the fitting procedure of the main text was applied to each processed experimental distribution, for each of the six days. Best-fit tra [file pcbi.1000952.s003.tif]

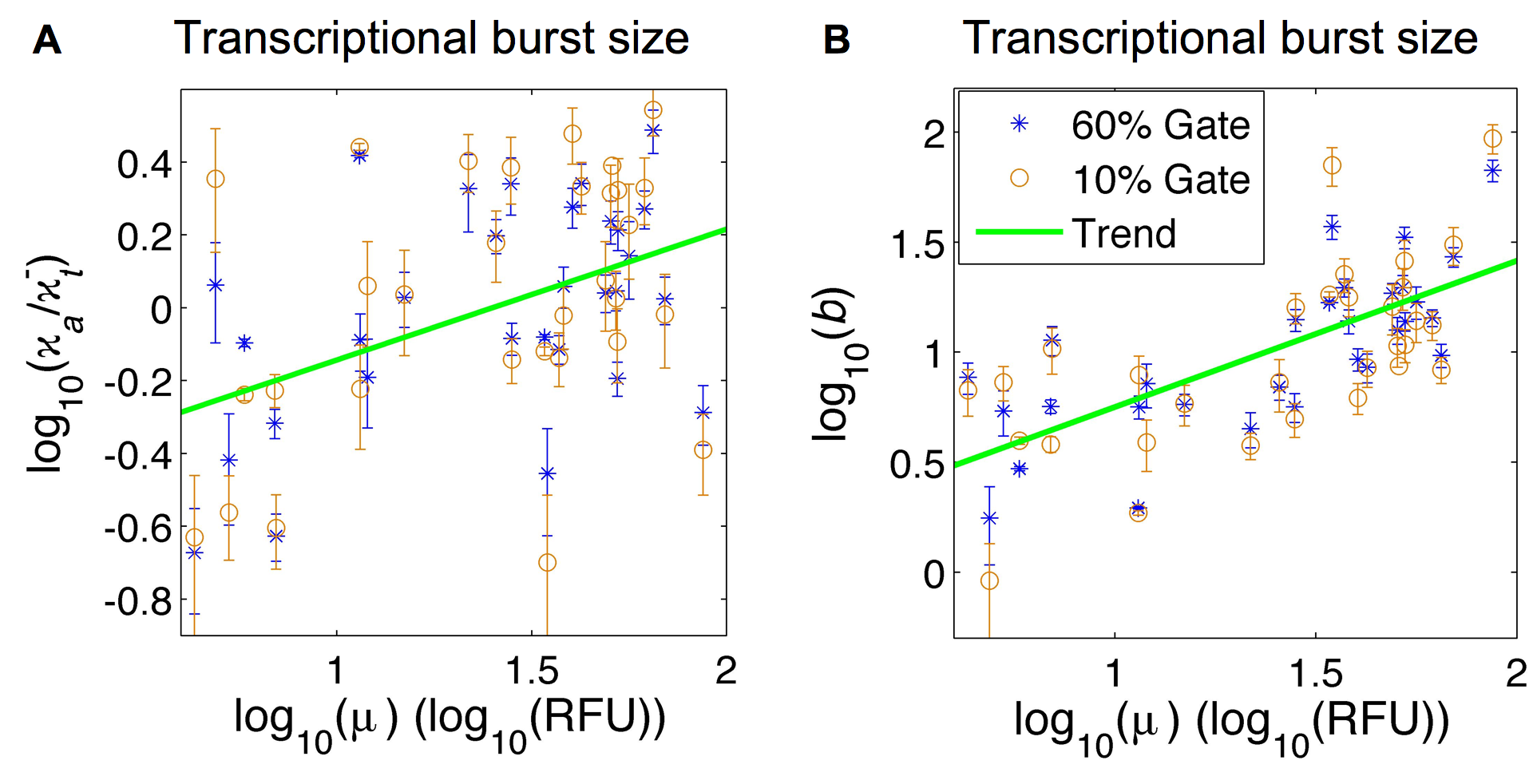

Supplement: Figure S4 — Gating for cell size does not significantly affect inferred trends in burst-parameter variation with integration position. A, B) The experimental distributions obtained by applying a 10% square gate in the FSC/SSC plane (as discussed in Sec. S.I.7) were fit following the procedure in the main text (‘narrow gate’), and the resulting best-fit model parameters compared to those obtained for each clone based on our optimized distribution processing procedure (‘optimal gate’, = 60%), that were given in Fig. 4. Bars represent 95% confidence intervals, as obtained in the main text. Fit parameters for the ‘narrow gate’ data only demonstrate slight differences from the ‘optimal gate’ data, and demonstrate no significant difference in trend with expression mean, confirming that our results are robust to gating for cell size. (0.55 MB TIF) [file pcbi.1000952.s004.tif]

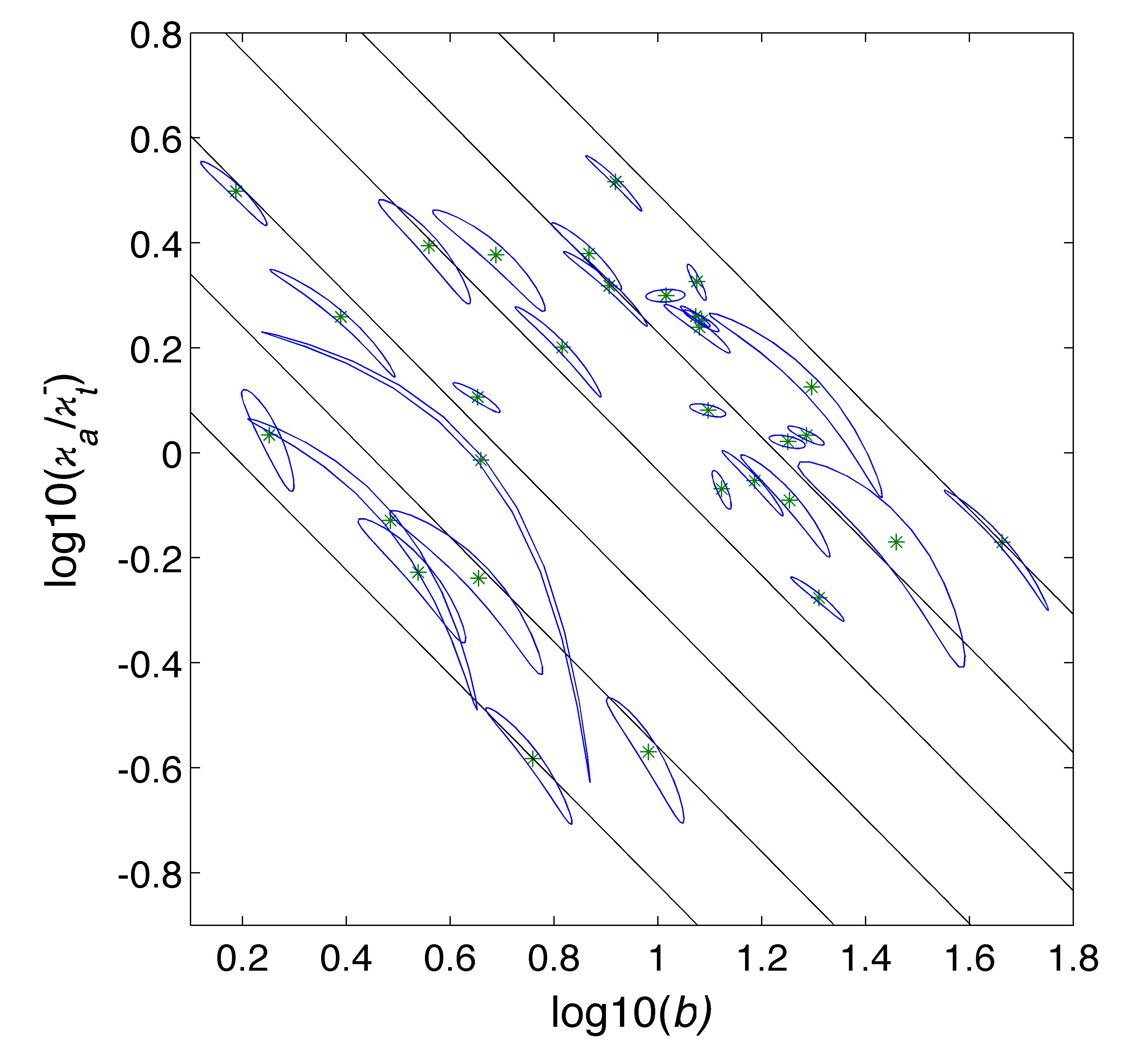

Supplement: Figure S5 — No significant correlation between transcriptional burst size and frequency for the HIV LTR. The best-fit transcriptional burst frequencies (κa), which were inferred for our system in Fig. 4 of the main text, are plotted against the corresponding inferred transcriptional burst size (b) for each clone (*) in a log-log plot to investigate possible correlations. Diagonal lines (green) represent combinations of burst size and frequency that specify constant mean expression (μ ∝ κa b in the bursting regime). The 95% confidence region calculated in the main text are ovals in the b×κa plane, and become deformed to rounded crescents in the log-log plot, represented by the closed curves about each combination of best-fit parameters (blue). Notice that in the region closest to the each best-fit parameter combination, the corresponding confidence boundary generally depicts less constrained variation in the direction that preserves expression mean (i.e. parallel to the drawn lines of constant mean). Linear regression reveals a slope of 0.17±0.3 (95% confidence), with an R2 value of 0.05, and a Pearson correlation coefficient of 0.2. We thus conclude that significant correlations between burst size and frequency, that might affect the trends in these parameters with distribution mean that were analyzed in Fig. 4 of the main text, are not present in our system. (0.35 MB TIF) [file pcbi.1000952.s005.tif]

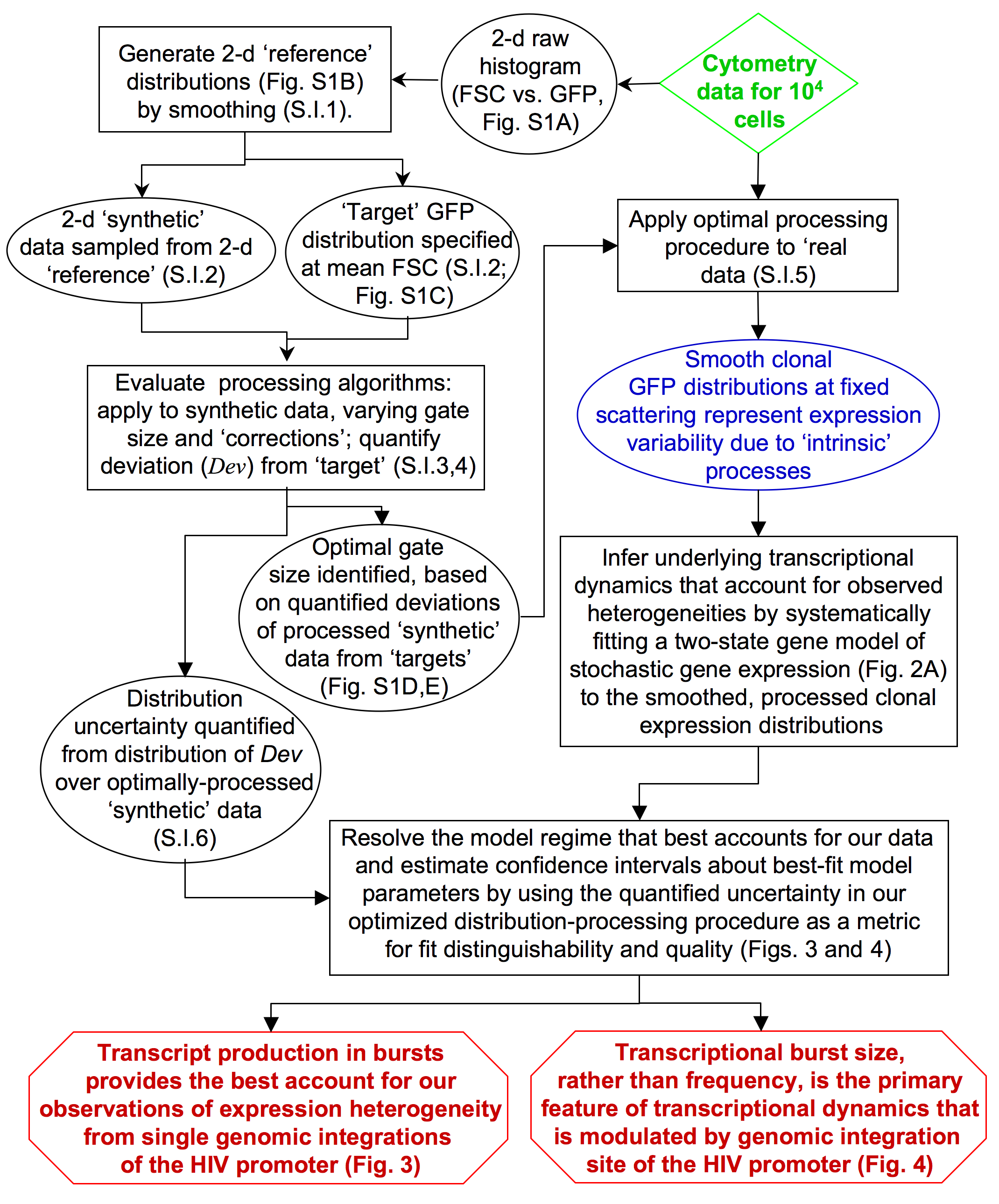

Supplement: Table S1 — Work flow. (0.97 MB TIF) [file pcbi.1000952.s006.tif]
