# Supplementary material for: HIV Promoter Integration Site Primarily Modulates Transcriptional Burst Size Rather Than Frequency
Source: PLoS Comput Biol. 2010 Sep 30;6(9):e1000952. doi: 10.1371/journal.pcbi.1000952 (PMC2947985; doi:10.1371/journal.pcbi.1000952)
Supplement: Text S1 — Supplement to “HIV-Promoter Integration Site Primarily Modulates Transcriptional Burst Size, Rather Than Frequency.” (0.89 MB PDF) [file pcbi.1000952.s007.pdf]

**SUPPLEMENT** to 'HIV-Promoter Integration Site Primarily Modulates Transcriptional Burst Size, Rather Than Frequency'

Authors: Ron Skupsky, John C. Burnett, Jonathan E. Foley, David Schaffer, Adam Arkin

**CONTENTS**

**I) Processing of cytometry data and assessing uncertainty in experimental distributions**

**II) Solving the model**

**III) Distribution moments**

**IV) Bursting regime**

**V) Estimating non-fit model parameters**

**VI) Fitting procedure and fit quality**

**VII) Summary of sources of uncertainty in data**

**VIII) Considering extrinsic sources of heterogeneity**

**FIGURES**

**Figure S1. Data processing**

**S1.A. Sample 2-d cytometry data**

**S1.B. ‘Reference’ smooth 2-d histogram**

**S1.C. ‘Target’ 1-d GFP slice**

**S1.D. Bootstrap estimate of distribution uncertainty for different sized gates**

**S1.E. Possible corrections to the processing procedure**

**S1.F. Fraction of expression variability due to correlation between GFP and FSC**

**S1.G. Scattering gate size has little effect on distribution ‘noise’**

**S1.H Scattering gate size has little effect on distribution skew**

**Table S1. Work flow diagram**

**Figure S2. Fit quality, improvement, and deviations**

**S2.A. Relative fit deviations**

**S2.B. Fit improvement for systematic search over moment fits**

**S2.C. Shape of the fit deviations**

**S2.D. Significance of fit-deviation shape**

**Figure S3. Distribution stability over time**

**S3.A. Fluctuations in clonal mean over time are uncorrelated**

**S3.B. Distribution-moment variation over time**

**S3.C. Sample distribution-shape variation over time**

**S3.D. Sample distribution time-variability after re-scaling**

**S3.E. Scatter in best-fit transcriptional burst frequency over time**

**S3.F. Scatter in best-fit transcriptional burst size over time**

**Figure S4. Scattering gate size has little effect on inference of transcriptional burst parameters**

**S4.A. Burst frequencies**

**S4.B. Burst sizes**

**Figure S5. Best-fit transcriptional burst sizes and frequencies demonstrate no significant correlation over the set of integration clones.**

## SI Text

### I) Processing of cytometry data and assessing uncertainty in experimental distributions

A number of studies have noted the importance of accounting for heterogeneity in cell size and cell cycle to resolve the component of expression heterogeneity that may be due to noise sources intrinsic to the biochemical processes involved in gene expression. In [1], this was accomplished by 'in-silico' synchronization of microscopy time courses to select cells whose growth had progressed through a specified fraction of the cell cycle since the previous division, as quantified by image-analysis-based size determination. In [2], where cytometry data similar to our own was analyzed (in yeast), it was found that gating the data to select a narrow range of forward and side scatter values (FSC and SSC, considered as measures of cell size and granularity respectively), that minimized the width of the fluorescence distribution over a clonal population of cells, and eliminated correlation between scattering measurements and cellular fluorescence, was an effective means of isolating the component of expression heterogeneity believed to be due to intrinsic expression noise (which was also quantified using a dual reporter system for several test cases in that study). Essentially, the effect of such a procedure is to select cells of uniform size and state. However, this procedure only uses a small fraction of the originally collected data. Thus, we wished to optimize a correction for our measurements that accounts for cell size and state in our quantification of expression heterogeneity, but which makes use of a larger fraction of the total cell population to improve statistics.

Our aim was to select an optimal method of distribution extraction that minimizes the uncertainty in inferring a protein expression distribution at a defined value of FSC, where we considered FSC measure as a proxy for counting cells of uniform size., and we chose the mean FSC to specify a fluorescence distribution, referring to this conditional GFP distribution as the 'target' distribution (i.e. the distribution that we aim to reproduce). We adopted a procedure that combines gating, possibly 'correcting' the data to further remove correlations, and smoothing the resulting histogram, and we used a bootstrap approach to quantify uncertainties in candidate processing procedures, which we outline below. Our procedure essentially balances the errors that are generated by: 1) counting cells whose scattering measures differ from the mean, and whose associated conditional GFP distribution may differ from the 'target' distribution (and we explore the possibly of correcting for these deviations), with 2) the increased counting error due

to only counting smaller numbers of cells within a very narrow gate of scattering measures, which was the procedure followed in [2]. Here we limit our discussion to optimization accounting for FSC variability. We note however, that we performed a similar analysis to account for variability in SSC, and found a similar gating to be optimal, with a similar quantification of distribution uncertainties. However, due to correlation between FSC and SSC, combining the two procedures had minimal effect on distribution shape, and our analysis of fit quality only includes uncertainty derived from the FSC procedure, which we now outline.

1) *A 'reference' distribution was generated for each clone*: Our goal is to optimize a procedure for estimating the conditional GFP distribution at fixed FSC (or SSC), e.g. for the set of cells with mean FSC values. To optimize a distribution-processing procedure, we began by specifying for each clone a 'reference' distribution – that is, a smooth 2-d distribution that captures the co-dependence of GFP and FSC, and might represent the underlying probability distribution from which the collection of cells in each experimental data set were generated. By re-sampling from these 'reference' distributions, we computationally generated a collection of 'synthetic' data sets (described below in S.I.2), with known underlying probability distribution, and known associated 'target' GFP distribution at fixed FSC, on which we could test and optimize different distribution processing procedures. Specifically, below we will identify an FSC (or SSC) gate size that, together with further distribution processing procedures to be discussed, when applied to a 'real' data set, such as that generated by cytometry measurements on $10^4$ cells from one of the clones in our study, produces an output distribution whose deviation from the 'target' GFP distribution at fixed FSC is minimized.

We generated 'reference' distributions by taking first the 2-d data set of GFP and FSC values for the $10^4$ cells that were initially measured for each clone, and generating a 2-d histogram for each (e.g. Fig. S1.A). We then applied a 2-d low-pass Fourier filter to generate a smooth 2-d distribution, which after normalization (i.e. the count in each bin was divided by the total number of cells counted for the histogram), we considered to reflect an underlying probability distribution that is 'close' to the actual underlying probability distribution of observing any combination of GFP and FSC values for a randomly selected cell for that clone (e.g. Fig. S1.B). Specifically, the filter was set by adjusting a single parameter specifying the cut-off in Fourier space, such that the resulting 2-d distribution specified a mono-modal GFP

distribution for any fixed value of FSC (i.e. the conditional distribution), and such that the mean GFP and FSC values, as well as their covariance matrix, were preserved to experimental accuracy. This resulted in a 'reference' 2-d distribution for each clone that quantitatively captured the dependence of the 1-d GFP (conditional) distribution on FSC measure, at least to linear order. The 1-d GFP distribution at the mean FSC slice through the 2-d smooth 'reference' distribution was then taken as the 'target' distribution that our processing procedure aims to reproduce for each clone (e.g. Fig. S1.C).

2) *Bootstrap-generated synthetic data*: We re-sampled from the smooth 2-d 'reference' distributions from step 1 to computationally generate 200 'synthetic' data sets for each clone, each of which included FSC and GFP values for $10^4$ cells; we considered a range of cell numbers to investigate whether the optimal procedure changed substantially if we took measurements on a different number of cells and found that it did not for a range of at least a factor of 10 about the number of cells counted in our experiments.

3) *Optimizing an FSC gate size*: We optimized our distribution-processing procedure by applying it to our 'synthetic' 2-d data sets, with the goal of reproducing their known 'target' 1-d GFP distributions at mean FSC for each clone. We first considered using different sized FSC gates. Each synthetic data set was gated to include only a fixed percentage of FSC values about the mean. The resulting GFP histogram for the gated cells was then normalized to generate a probability distribution, and a 1-d Fourier filter was applied to generate a smooth distribution (the 1-d Fourier cut-off was optimized to reproduce a smooth underlying 1-d probability distribution from any given number of sampled cells, based on a bootstrap procedure similar to that outlined here). For each size gate, for each clone, the distribution-deviation after gating and smoothing, of each 'synthetic' data set from the 'target' distribution (*Dev*, see Fig. S.1 caption and Materials and Methods of the main text for definition), was calculated and then averaged over the set of synthetic data sets (Fig. S1.D). This average deviation provided a measure of the error in estimating the 'target' distribution, from an experimental distribution of the considered type, by applying a given gate size, for each clone.

We found that for our experimental data sets, for most clones, the optimal gate size for identifying a GFP probability distribution at the mean FSC value, included cells whose FSC

values spanned approximately the mid 40-60% of values. This was the optimal balance between improving counting statistics and selecting cells of uniform size (a similar result was found for gating SSC values). Apparently, over this range of FSC values, the effect on the GFP distribution of including larger and smaller cells effectively cancel each other. This finding is consistent with the observation that the expression distributions in our study are much 'noisier' than most that had been analyzing in other studies [1,2], with cellular fluorescences spanning a larger than typical range of values relative to the mean fluorescence for any given clone. This is discussed further below in Section S.VIII where we address extrinsic source of noise'; in the Discussion section of the main text we considered features of the HIV promoter that might account for its observed large expression variability, and how this variability might play an advantageous role in the viral life-cycle dynamics.

4) *Possible 'corrections' to the processing procedure:* Two other possible 'size corrections' were considered that might allow us to further increase the range of FSC values included in our histograms for our processing procedure to optimally resolve a 'target' GFP distribution at fixed FSC. We followed the same procedure as in 3. But subsequent to gating, we either subtracted linear correlation between FSC and GFP from the GFP values ('*lin*'), or divided by the ratio fo the FSC value to the mean FSC value ('*div*'). In each case, we found that agreement with our 'target' distribution was not significantly improved, and a gating window of order 60% remained optimal (Fig. S1.E).

5) *Applying the optimal procedure to the 'real' data*: Based on our optimization of a distribution-processing procedure for our 'synthetic' data (which was re-sampled from the original data after smoothing, see S.I.2), we followed the simple 'gating' procedure identified above in S.I.3 to process our experimental data, keeping only cells whose FSC values spanned the mid 60% for each clone. In this way, we optimally extracted a smooth GFP distribution that represents the probability of observing a given cellular fluorescence in cells whose FSC value is fixed at the mean over the population. A similar procedure was applied to select cells whose SSC values span the mid 60% (which was found as well to be nearly optimal for selecting a GFP distribution at the mean SSC for all clones). As a result of gating by both FSC and SSC, data

from approximately 3600 cells per clone were used to generate the final histograms that were smoothed and used in our analysis for model fitting.

6) *Estimate of uncertainty in our experimental distributions*: The analysis in Step 3 then provides an estimate of the uncertainty in estimating a target GFP distribution at the mean FSC value by using a gate that spans the mid 60%. In other words, the distribution of deviations for our optimized processing procedure, between the processed 'synthetic' data sets and their associated 'target', for each clone (both specified by the 'reference' distribution for each clone), provides an estimate of the deviation that we expect between our optimally processed experimental data and the actual underlying probability distribution of observing a GFP value from that clone for a cell whose FSC value is the average over the population. Specifically, we used the 95% upper bound on the values of distribution deviations (*Dev*) from the 'target' over the set of 'synthetic' data sets for each clone as a measure of the uncertainty in identifying the desired underlying probability distribution from our cytometry data. This estimate of uncertainty in our experimental distributions was then used to normalize the calculated deviations between our model fits and the processed experimental distributions, as described in the Materials and Methods section of the main text.

7) *Quantifying the contribution of cell-size variability to expression variability*: As a check on the contribution of cell-size variability to our measurement of GFP expression variability, we calculated the relative contribution to the GFP distribution variance from correlation between GFP and FSC measurements (Fig. S1.F). This quantity is essentially the $R^2$ value for linear regression of fluorescence measurement against FSC. In general, if we take FSC as a measure of cell size, we conclude that cell-size variability only contributes a few percent of the observed GFP distribution variances, despite the wide gate of FSC values that we have adopted. This suggests that the large expression heterogeneities observed in this study are not due to variability in cell size (see Sec. S.VIII for further discussion).

As an extra check, to be sure that our wide gating is not distorting the shape-features of our processed distributions, we considered the behavior of distribution coefficients of variation ('CV' = $\sigma/\mu$) and skewness ('skew' = $m_3/\sigma^3$, where $m_3$ is the distribution 3rd central moment), for varying gate sizes in the FSC/SSC plane (Figs. S1G-H). We find that even at gate sizes as small

as 10% (i.e. a factor of 6 smaller in each scattering dimension than the optimized gate discussed above), the coefficient of variation is only typically affected by a few percent, and the skew by approximately 15%. Further, if we normalize our GFP values by subtracting off the average correlation between GFP and FSC (or SSC), as suggested above in Sec. S.I.4, the decrease in average coefficient of variation and skew at smaller gates is approximately cut in half (not shown). In general, the effect on distribution moments of reducing the gate size to 10% demonstrates significant scatter, and a number of clones surprisingly demonstrate larger coefficients of variation and skews for reduced gate sizes. This analysis further confirms that our relatively wide 60% gating is only generating a slight overestimate of distribution noise and skew, and that our choice of gate size is warranted as optimally reproducing a GFP distribution at a fixed value of scattering measure.

The approach taken here to optimize out distribution-processing procedure, and the role it plays in our analysis of heterogeneity-generating transcriptional dynamics, is summarized in a flow diagram, Table S1.

**II) Solving the model**

The model in Fig. 2A was solved numerically to identify the model-predicted probability of observing a given number of copies of protein (GFP) in a given cell, for comparison to our processed experimental distributions. To systematically search the parameter space of the model and identify a best-fit parameter combination for each clone, an efficient and precise algorithm was necessary. The algorithm that was used, which we discuss below, was implemented in Matlab (The Mathworks) and is available upon request.

The model in Fig. 2A of the main text represents a Markov process, where all rates denote a probability per unit time. The evolution of the probability distribution function (PDF) denoted $W\{\phi, m, n\}$ – which specifies the probability of observing a cell in a particular configuration defined by the gene-state ($\phi_{a/r}$), the number of copies of the transcript present in the cell ($m$ copies), and the number of copies of the protein ($n$ copies) – is given by the Chemical Master Equation (CME) for the system, which is a standard representation for describing well-mixed chemically reacting systems [3]. Marginalizing over the gene-state and transcript number

distributions gives the protein number distribution that we wish to calculate to compare with our experimental cytometry data.

The CME for our system is:

$$\frac{dW\{\phi_{a/r},m,n\}}{dt} = -\left(\kappa_{r/a} + \kappa_t^+ + \kappa_t^- m + \kappa_p^+ m + \kappa_p^- n\right)W\{\phi_{a/r},m,n\}$$
$$+\kappa_{a/r}W\{\phi_{r/a},m,n\} + \kappa_t^+ W\{\phi_{a/r},m-1,n\} + \kappa_t^-(m+1)W\{\phi_{a/r},m+1,n\} \qquad (1)$$
$$+\kappa_p^+ mW\{\phi_{a/r},m,n-1\} + \kappa_p^-(n+1)W\{\phi_{a/r},m,n+1\}$$

The full set of equations specified by eq. 1, for the probability of observing any state, is an infinite linear system of ordinary differential equations (ODEs).

For many-component systems (i.e. large numbers of reacting species), a direct solution to the master equation is not computationally feasible, and stochastic simulation techniques are often used to directly simulate trajectories of the stochastic process under consideration [4], with a large number of simulations generally necessary to sample the desired underlying probability distribution. However, for systems such as ours, with only a few components, a direct solution to the CME becomes feasible, and it can provide a fast and accurate method for calculating the desired probability distributions. This is the approach that we took.

Though an analytic solution exists for the transcript distribution in our model, as well as for a small number of other simple models [5,6,7], none exists for the protein distribution in our model. We therefore solved the system numerically. Here we discuss several important aspects of the numerical algorithm that we used. In particular: 1) Truncating the system at large protein and transcript numbers; 2) A continuum approximation that we used to coarse-grain the distribution; 3) The algorithm that was used to evolve the system in time to steady state; 4) Several checks that were used to ensure that the algorithm converged to the correct distribution.

1) *State-space truncation*: In order to numerically solve the infinite system of equations represented by the CME, it was necessary to truncate the system at large values of transcript and protein number to specify a finite set of equations. We chose to truncate the system at transcript and protein numbers with values corresponding to the steady-state mean plus 15X the standard deviation of the transcript and protein distributions, which we analytically calculated (see Sec. S.III below). All probabilities were set to zero beyond these cut-offs, which represent states of

the system that are effectively 'almost never' visited by the system over the time-course of our simulations.

The consistency of this choice of cut-off was confirmed in two ways. First, for all test cases where we examined them, the calculated probabilities of observing states at the boundary of our cut-off were near the order of the machine precision. Second, the Finite State Projection (FSP) algorithm, developed elegantly by Munsky and Khammash [8], provides a rigorous method of calculating an upper bound on the error due to state-space truncation. We applied this procedure to several test cases where the system was small enough to be solved exactly by the FSP, and always found the error-bound to be several orders of magnitude smaller than the uncertainty in our experimental distributions, over the mid 98% of the distribution.

2) *Coarse-graining*: The CME is an exact formulation to evolve the probability of observing each individually-enumerated state of a chemically reacting system. However, for systems with large numbers of particles, the CME represents a very large system of equations (even after truncation). Under these conditions, though the system is large, the desired distributions often vary smoothly and admit a continuum approximation. For example, a system-size expansion leads to a Fokker-Planck Equation (FPE) [3,9], which for our system would represent transcript and protein numbers as continuous variables, and their evolution would be described by a pair of Partial Differential Equations (PDEs), rather than an infinite system of ODEs. For systems with large numbers of particles but small numbers of components, a numerical solution to the FPE can often be achieved with acceptable accuracy more efficiently than the solution to the original CME – that is, if a discretization can be achieved that leads to a substantially smaller number of equations through appropriate approximations of the PDF derivatives that are involved in the FPE. On the other hand, for systems that may sample both states of high and low particle numbers, as is often the case for cellular systems and in particular for our own system, relevant regions in the state space exist where a continuum approximation can provide an accurate computation, while other regions exist containing states of low particle number where a continuum approximation is inaccurate and it is necessary to solve the master equation to achieve acceptable accuracy in determining the PDF. For such systems, hybrid algorithms are often employed to partition the system and apply an appropriate and efficient method in each part of the state space and/or to different subsets of reactions. Such hybrid algorithms have generally

been developed in connection with stochastic simulation algorithms [4,10,11,12,13], while methods that directly use a continuum approximation, such as the FPE, might use a state-space discretization for their numerical algorithm that converges to the CME for small particle numbers [13].

To take advantage of a continuum approximation at large particle numbers in our system, while keeping an exact CME treatment at low particle numbers, we applied a graded coarse-graining procedure. Probabilities were binned together, separately for each gene-state, into 'rectangles', or 'bins' in the transcript-protein plane. Bin sizes increased for larger transcript and protein numbers, where the distributions generally changed more slowly and were approximated well by a continuous distribution that could be expanded in a Taylor series for small changes in protein and transcript number. An approach to grading that we found to yield efficient and stable solutions was coarse-graining with bin sizes increasing in proportion to the square root of the protein or transcript number, in such a way that bins with protein or transcript numbers below 50 included exactly 1 state (any number at least of order 10, below which we required single-state bins, was effective in this respect).

The probability in a 'bin' was specified as the sum of the probabilities of all system configurations in the bin. Transition rates between 'binned' configurations were then specified by linearly approximating the values of the distribution at the boundaries of the bins. Thus, transition rates from one gene-state to another were still calculated exactly, while protein and transcript transitions were accounted for based on this continuum description, whose coarseness increased for larger transcript and protein numbers. For states of small transcript and protein number, this method retains the exact form of the CME. For large transcript and protein numbers, the resulting equations are equivalent to a discretization of the FPE that corresponds to our system. Coarse-graining and interpolation for transition rates was done in such a way that retains the probability-conserving feature of the CME.

This approximation scheme greatly increased computation speed, and its validity was checked by increasing and decreasing the length-scale of the coarse-graining/binning procedure by a factor of 2, over which the solutions were found to remain stable.

3) *Evolving the system to steady state*: The CME for our system, as well as the smaller system that resulted from our coarse-graining procedure, represents a stiff system of ODEs. For

this reason, the system is integrated more efficiently with an implicit solver (one that evaluates derivatives based on the function values at the next time step for which it is solving). A method that we found to be fast and stable steps through time by treating, on a given time step, the transitions due to transcript and gene-state dynamics implicitly (using a backwards Euler method) and those due to protein dynamics explicitly (using a forward Euler method), and exchanging the terms that are treated implicitly and explicitly in the following time-step. Such methods are used to integrate multi-dimensional PDEs, in order to maintain numerical stability while taking longer time steps, and they facilitate the use of fast matrix inversion methods that take advantage of the banded structure that arises when only a single dimension is treated implicitly at each time-step in reaction-diffusion type problems [14].

The system was initialized with the gene inactive and no transcript or protein present ($W\{\phi_r, 0, 0\} = 1$). The system was then integrated by the above procedure till a steady-state distribution was reached. Steady state was determined when the relative change in probability for any bin with value greater than 0.1% of the maximum over the set of bins (this generally covered more than the mid 98% of protein values, which we used to compare to our experimental distributions) fell below $10^{-5}dt$, where $dt$ is the size of the time step. This represents a change 4 orders of magnitude slower than the slowest time scale in the problem (which was generally the protein degradation time), and the total time for which the system was evolved was always at least 50X the protein degradation time. This steady-state criterion always resulted in good agreement between the moments of the calculated distribution and their theoretical steady-state values (see below)

4) *Solution accuracy:* We checked the accuracy of our calculations by multiple methods, including the checks on the various approximations in our algorithm that were mentioned above. In addition, we compared the first 3 moments of our simulated distributions to their theoretical values, where the distribution moments were analytically calculable as outlined below in the next section. Deviations were always less than 0.1% (this value is generally less than the uncertainty in these moments estimated for our experimental data), and often significantly less. In addition, for several test cases, where the mean transcript and protein numbers were small enough to calculate an effective steady state distribution by the FSP algorithm without any approximation – this is an exact calculation with an associated rigorously-calculated error bound [the FSP was

discussed above; see 8] – we compared our results to this method. We typically found relative deviations between our solution and the FSP solution to be several orders of magnitude less than the uncertainty in our experimental distributions.

**III) Distribution moments**

Distribution moments of all orders can be obtained analytically for our system by standard methods [3], and were important for qualitatively analyzing our experimental distributions, characterizing the bursting regime in our theoretical analysis, generating initial guesses at best-fit model parameters, and specifying non-fit model parameters from independent measurements. Here we use a generating-function approach to calculate the distribution moments of the model. The generating function for the system is defined as:

$$F_{a/r} = \sum_{m,n} W\{\phi_{a/r}, m, n\} x^m y^n \tag{2}$$

and satisfies the generating-function equation:

$$\begin{aligned} \frac{\partial F_{a/r}}{\partial t} &= -\kappa_{r/a} F_{a/r} + \kappa_{r/a} F_{r/a} + \kappa_t^+ (x-1) F_{r/a} - \kappa_t^- (x-1) \frac{\partial F_{r/a}}{\partial x} \\ &+ \kappa_p^+ (y-1) F_{r/a} - \kappa_p^- (y-1) \frac{\partial F_{r/a}}{\partial y} \end{aligned} \tag{3}$$

This is simply a restatement of the infinite system in eq. 1 in multivariate form, and the full set ODEs that specifies the Master Equation for the system can be recovered by equating coefficients of powers of $x$ and $y$ on both sides of eq. 3.

Moments of the distribution are calculated by taking derivatives of eq. 3 with respect to $x$ and $y$, and evaluating at $x = y = 1$. In particular the mean number of transcripts and proteins are given, respectively, by

$$\mu_T = \langle m \rangle = U_x\big|_{x=1,y=1} \equiv \left.\frac{\partial U}{\partial x}\right|_{x=1,y=1}, \quad \mu_P = \langle n \rangle = U_y\big|_{x=1,y=1}, \quad U = F_a + F_r \tag{4}$$

where the bracket, $\langle\ \rangle$ denotes an average over all gene states, transcript numbers, and protein numbers, for the probability distribution generated by the two-state model. At steady state, this yields the expression that would be expected from assuming deterministic, mass-action kinetics. Namely:

$$\mu_T = \langle m \rangle = \frac{\kappa_t^+}{\kappa_t^-} \frac{\kappa_a}{\kappa_a + \kappa_r}, \quad \mu_P = \langle n \rangle = \frac{\kappa_p^+}{\kappa_p^-} \mu_T \tag{5}$$

In general, one can generate a hierarchy of algebraic equations to be solved for higher derivatives of the generating function with respect to $x$ and $y$, which are used to calculate higher moments of the distribution, in terms of lower derivatives, and eventually in terms of model parameters. This allows one to calculate analytic expressions for all moments of the joint distribution for the system in terms of model parameters. In particular, variances of the protein and transcript distributions are given according to:

$$\sigma_T^2 = \left\langle (m - \langle m \rangle)^2 \right\rangle = \langle m^2 \rangle - \langle m \rangle^2, \qquad \sigma_P^2 = \left\langle (n - \langle n \rangle)^2 \right\rangle = \langle n^2 \rangle - \langle n \rangle^2, \tag{6}$$

$$\langle m^2 \rangle = \frac{\partial}{\partial x} x \frac{\partial U}{\partial x}\bigg|_{x=1,y=1} = \left(U_{xx} + U_x\right)\Big|_{x=1,y=1}, \qquad \langle n^2 \rangle = \left(U_{yy} + U_y\right)\Big|_{x=1,y=1},$$

and at steady state we find:

$$\sigma_T{}^2 = \frac{\kappa_a \kappa_r \kappa_t^-}{(\kappa_r + \kappa_a)^2 (\kappa_r + \kappa_a + \kappa_t^-)} \left(\frac{\kappa_t^+}{\kappa_t^-}\right)^2 + \mu_T \tag{7}$$

$$\sigma_P{}^2 = \mu_P + \frac{\kappa_p^-}{\kappa_p^- + \kappa_t^-}\left(\frac{\kappa_p^+}{\kappa_p^-}\right)^2 \sigma_T{}^2 + \frac{\kappa_t^-}{\kappa_p^- + \kappa_t^-}\frac{\kappa_p^-}{\kappa_a + \kappa_r + \kappa_p^-}\left(\frac{\kappa_p^+}{\kappa_p^-}\right)^2 \left(\sigma_T{}^2 - \mu_T\right) \tag{8}$$

The same expressions for the first two moments of the protein distribution for this model are given in slightly reorganized form in the supplement to [15], and the first two moments of the transcript distribution are given, together with a very elegant analytic derivation of the full transcript distribution, in [5].

As mentioned in Sec. S.II, though all distribution moments for the two-state model are analytically calculable, the protein distribution its self is not analytically calculable, and was solved for numerically as described above. The first 3 analytic moments were therefore used to assess both the accuracy of our numerical simulations, and convergence to steady state.

## IV) Bursting regime

The bursting regime is specified by relatively short active-state durations ($\kappa_r >> \kappa_t^-$), multiple transcripts produced during each gene-activation event ($b = \kappa_t^+/\kappa_r$ finite), and moderate frequency of gene activation (in particular, $\kappa_r >> \kappa_a$). Under these conditions, the distribution mean and variance reduce to:

$$\mu_T \sim \left(\kappa_a / \kappa_t^-\right) b, \quad \mu_P = \frac{\kappa_p^+}{\kappa_p^-} \mu_T \ (as\ before) \tag{9}$$

$$\sigma_T{}^2 \sim \left(\kappa_a / \kappa_t^-\right) b^2 + \mu_T = (b+1)\mu_T \tag{10}$$

$$\sigma_P{}^2 \sim \mu_P + \frac{\kappa_p{}^-}{\kappa_p{}^- + \kappa_t{}^-}\left(\frac{\kappa_p{}^+}{\kappa_p{}^-}\right)^2 \sigma_T{}^2 \tag{11}$$

The active-duration for the two-state model is the average time that the gene remains in the active configuration during a single visit, and is given by:

$$\tau = 1/\kappa_r \tag{12}$$

(the distribution of lengths of time for a single visit to the active state is exponential, with decay constant $\kappa_r$, and here we omit the normalization by transcript decay time that is used in the main text and model fitting). While the gene remains in the active state, transcript is produced at a fixed rate (i.e. in a Poisson process), and the average number of transcripts produced during a single visit to the active state is given by the product of the production rate ($\kappa_t^+$) and the average time that the gene remains in the active state (i.e. $\tau$). Namely:

$$b = \kappa_t^+\tau = \kappa_t^+/\kappa_r \tag{13}$$

This is the quantity that we have defined as the transcriptional burst size. For any regime of the model, this number specifies the average number of transcripts produced during a single visit to the active state. For relatively long active-durations, a number of transcripts will be degraded as well during a typical visit of the system to the active configuration, such that the full transcriptional burst will not be observed in any cell at a single time. However, in the 'bursting' regime, active-durations are short, and the transcriptional burst size quantifies the number of new transcripts expected to be present in the cell at the time when the system returns to the inactive gene-state, i.e. immediately after a completed single visit to the active configuration.

The frequency of gene-activation events is specified by the reciprocal of the average time for a cycle of activation and inactivation, i.e. by the reciprocal of the sum of the average time between one gene activation event and the following inactivation event plus the average time between an inactivation event and the following activation event. The average time spent during a visit to the inactive state is given as $\tau_r = 1/\kappa_a$. Thus, the frequency of activation events is given by $1/(\tau + \tau_r)$ = $\kappa_a \kappa_r / (\kappa_a + \kappa_r)$. In the bursting regime, the frequency of gene-activation

events reduces to $\kappa_a$, because $\kappa_r >> \kappa_a$. Thus, in the bursting regime, $\kappa_a$ specifies the burst frequency, and the mean transcript number is given by the product of burst size and burst frequency (normalized by the rate of transcript degradation, see eq. 9).

**V) Estimating non-fit model parameters**

A number of the processes represented in Fig. 2A of the main text likely occur at locations that are spatially separated from the site of viral integration in our system, and are therefore expected to occur at the same rate for all integration clones. Namely, the rates of transcript degradation ($\kappa_t^-$), and protein production ($\kappa_p^+$) and degradation ($\kappa_p^-$), are assumed fixed over the full set of integration-clones. A conversion factor from protein number to cytometer-measured relative fluorescence units (RFU) is also necessary to compare model results to our experimental data. We will refer to this factor as '$\alpha$'. Here we discuss how these parameters were measured experimentally

Protein degradation in our model should more precisely labeled as protein dilution – because of the slow time-scale of GFP degradation, the process that effectively decreases the concentration of GFP in the cell is growth, which increases the cellular volume (of course the cell must produce GFP in excess of the 'actual' degradation rate in order to maintain a constant concentration in the face of cell growth, at steady state). Thus, the cell growth-rate defines an 'effective' GFP degradation/dilution rate. The cellular growth-rate was measured by adding a non-degraded fluorescence marker to label the cells (Cell-Trace DDAO-SE Far Red, Invitrogen), and flow cytometry was used to measure single-cell fluorescence and generate population fluorescence histograms over time. A decay curve yielded a GFP dilution time of approximately 20 h. The absolute value of this rate is not essential for our analysis because, as mentioned in the main text, at steady state only the relative values of the rates in our model affect the model distributions. However, having a measured value of this rate allows us to calculate absolute values of our other model parameters from the relative values inferred by our analysis.

In order to calibrate the remaining non-fit model parameters, we used data from preliminary transcript-counting measurements using fluorescence in-situ hybridization (FISH) (Foley et al., *manuscript in preparation*). This allowed us to compare the mean and variance of the transcript distribution for the analyzed clone to the mean and variance obtained from the

cytometry-based protein distributions. The RFU mean is related to the transcript mean according to the relationship:

$$\mu_{RFU} = \left( \alpha \frac{\kappa_p^+}{\kappa_p^-} \right) \mu_T \qquad (14)$$

via eq. 5. We thus obtained $\alpha\left(\kappa_p^+ / \kappa_p^-\right) = 2.5$ from the measured ratio of population-mean RFU to population-mean transcript number for the analyzed clone. Similarly, in the bursting regime the distribution variances are related according to eq. 11, and the 1st term on the RHS can be dropped in the regime of large protein numbers, yielding:

$$\sigma_{RFU}{}^2 \sim \frac{\kappa_p{}^-}{\kappa_p{}^- + \kappa_t{}^-} \left( \alpha \frac{\kappa_p{}^+}{\kappa_p{}^-} \right)^2 \sigma_T{}^2 = \frac{1}{1 + \kappa_t{}^- / \kappa_p{}^-} 6.25 \sigma_T{}^2 \qquad (15)$$

after regrouping terms and using and the above-estimated value of $\alpha\left(\kappa_p^+ / \kappa_p^-\right)$. From the measured ratio of RFU and transcript variances for the considered clone, we thus obtained $\kappa_t{}^- / \kappa_p{}^- = 4$. Using the measured value of transcript dilution rate, this yields $\kappa_t^- \sim 5h$.

Because protein numbers are large in our system, their fluctuations do not significantly affect the expression profile. At steady state the model distributions depend on the protein production rate only through a simple scaling of all fluorescence values by the ratio $\kappa_p^+ / \kappa_p^-$, which in converting to measured RFU values becomes the ratio $\alpha\left(\kappa_p^+ / \kappa_p^-\right)$. i.e. the protein production rate and conversion factor to cytometry-based RFU only enter into our analysis in this combination. The measurements discussed above thus specify all of the integration-independent model parameters necessary for our analysis, together with our choice of a protein production rate in the regime where model-predicted typical protein numbers are large (protein numbers of order hundreds, or even tens, are sufficient to be in the regime where the actual value of $\kappa_p^+ / \kappa_p^-$ does not significantly affect distribution shape, and we chose $\kappa_p^+ / \kappa_p^- \sim 20$ for convenience, noting that the experimental values for our system are likely several orders of magnitude greater, e.g. using the average value of $\kappa_p^+ / \kappa_t^- \sim 1200$ estimated by [16] over their data set gives an estimate of $\kappa_p^+ / \kappa_p^- \sim 4800$ for our system).

The effects of uncertainties in the values of our non-fit model parameters were not explicitly considered in our analysis in the main text. Here we comment on how these

uncertainties might affect our findings. Uncertainties in determining the ratio $\alpha\left(\kappa_p^+/\kappa_p^-\right)$ were analyzed by sampling several values above and below the measured value (differing by up to a factor of 2.5), and then re-applying our fitting procedure to the cytometry data. Variations in $\alpha\left(\kappa_p^+/\kappa_p^-\right)$ were found to proportionately effect our determination of transcriptional burst size, though this proportionately was only partially maintained for dimmer clones (not shown). The effects of uncertainty in the ratio $\kappa_t^-/\kappa_p^-$ were similarly assessed by sampling several values about the measured value, and were found to proportionately affect the transcriptional burst frequencies inferred by our fitting procedure (not shown). Thus, uncertainties in non-fit model parameters do affect the absolute values of the inferred integration-dependent model parameters in our system. But these uncertainties affect all clones proportionately, and we thus expect that the trends in model-inferred dynamics over the set of viral integrations would be unaffected. i.e. our key finding, that transcriptional burst size is the primary feature that varies over viral integrations, is expected to be robust towards uncertainty in non-fit model parameters.

**VI) Fitting procedure and fit quality**

The routine that we used to search the model parameter space for a best-fit combination for each clone, which is discussed in the main text, required a parameter estimate for initialization. This initialization was accomplished by estimating model parameters based on the first two distribution moments, and assuming transcriptional bursting, as follows.

In the bursting regime, distributions are effectively described by the transcriptional burst size and burst frequency (with other non-fit parameters fixed at their separately measured values, see Sec. S.V above). More generally, for a fixed value of active-state duration ($\tau$), the model is specified by only two integration-dependent parameters (in any regime), which reduce to the transcriptional burst size and frequency in the bursting regime. Since the mean and variance are analytically calculable for any combination of parameters (see Sec. S.III above), their analytic forms (given by eqs. 5, 8) could be inverted to specify the remaining integration-dependent model parameters in terms of the distribution moments. Thus, we were able to analytically calculate a set of model parameters that reproduce the first two moments of each experimental distribution, for any fixed active duration, for each clone. In the bursting regime, this led to an analytically calculable transcriptional burst size and burst frequency. These calculated 'moment-

fit' parameters were used to initialize the more systematic fitting routine that was used to obtain the results discussed in the main text. To be certain that the results of our fitting procedure were not dependent on the moment-based initial guess of the fit parameters, the initial-guess parameters were varied randomly by up to a factor of two, and the routine always converged to the same set of best-fit parameters for each clone, to within our estimate of experimental accuracy.

The quality of the final fits was such that the deviations from the experimental distribution (*Dev*) for a number of clones were comparable to the uncertainty in our data (Fig. S2.A), and most were significantly improved over the initial moment-based guess (Fig. S2.B). In particular, the moment-fits generally did not account for the full skew of the experimental distributions, while the more systematic fitting routine, which minimized the deviation between the full model and experimental distributions (as described in the main text), only approximated the mean and variance, allowing small deviations in these features in order to better capture other distribution features such as skew. Nevertheless, some systematic deviation in the fits remained, with the systematic model fits still often demonstrating smaller skews than the data. That is, model fits often peaked to the right of the data and still underestimated the right tail of the distribution at larger fluorescence (Fig. S2.C). The scale of the distribution deviations was such that for the portions of the distribution where they were maximal, their magnitude generally remained larger than expected due to uncertainty in the data, even for fits where the overall deviation (*Dev*) fell below the value expected due to experimental uncertainty (Fig. S2.D). Investigation of other processes that could be added to the model to account for these deviations will be the subject of a future study.

**VII) Summary of sources of uncertainty**

The primary sources of uncertainty considered in our analysis are counting error (due to the finite number of cells counted to generate our experimental histograms), and error due to uncertainty in specifying a fluorescence distribution at fixed values of scattering measurements, as outlined above in section S.I.

Another potentially important source of uncertainty in our measurements is distribution drift over time, since our analysis assumes that our measurements represent steady-state expression profiles. We carried out longitudinal studies on 6 clones, where GFP expression

profiles were measured daily by cytometry over the course of approximately one week. We found expression profiles to be relatively stable over time, with mean expressions fluctuating by a few percent, and no significant correlations between clones (Fig. S3.A). However, some systematic drift was evident in the processed distributions. The primary affect of this drift on distribution shape was to effectively scale all fluorescence values by a factor of 1 ± a few percent, such that the distribution were effectively shifted with their shapes preserved over the log-binning of our cytometry measurements (for a log-binned histogram, a distribution scaling effectively translates the distribution by a fixed number of bins, while maintaining the distribution shape). For small variations, such a distribution scaling implies that the relative change in the distribution variance is proportional to twice the relative change in distribution mean. This proportionality is clearly seen for our data, and is demonstrated in Fig. S3.B. Indeed, distribution variability from day to day appeared qualitatively as a translation on the log-binned fluorescence axis for the plotted fluorescence histograms, a sample of which is given in Fig. S3.C. Further, for each clone, scaling the fluorescence values for distributions taken on different days by the ratio of their means effectively eliminates a substantial fraction of the day-to-day distribution variability. Such rescaled distributions demonstrate much smaller variability (e.g. Fig. S2.D), such that the remaining distribution drift over time was often on the order of that expected due to the distribution-processing errors quantified in Sec. S.I.

The fact that no correlation was found between the daily fluctuations in distribution mean between the clones (i.e. Fig. S3.A) suggests that distribution drift over time is not due to drift in our instrumentation. A distribution scaling, such as that which characterizes our longitudinal measurements, might arise for example if cellular transcription or translation rates were to drift over time, perhaps due to slight differences in cell density or to other small differences in the growth conditions for each clone, and might be incorporated in our analysis as uncertainties in these parameters. Theoretical analysis of the propagation of experimental uncertainties to our inference of best-fit model parameters suggests that such uncertainties in cellular transcription and translation rates lead to a proportional scaling of the transcriptional burst size identified by our fitting procedure for each clone (e.g. as discussed above in Sec. S.V.). A few percent variation in distribution mean due to drift over time translates into uncertainty of a few percent in the inferred transcriptional burst size. Such an uncertainty would not be correlated over the set of clones, and its magnitude is on the order of the uncertainties that we have currently identified

due distribution-processing error or smaller. Thus, such an uncertainty would not significantly affect the inferred trends in burst-parameter variation over the set of integration clones.

To confirm that distribution drift over time would not significantly affect our model-based inference of the underlying transcriptional dynamics, we applied our fitting procedure to the processed experimental distributions taken on each of the six days, for each clone sampled in our longitudinal study. In Fig. S3.E-F we plot the calculated best-fit transcriptional burst frequencies (S3.E) and burst sizes (S3.F) for each clone, for each day, relative to their time-average, against the log expression mean. Heavy bars about the value of 1 represent 95% confidence intervals for a single fit of a given clone as calculated in the main text, which do not include uncertainty due to distribution variability over time. We find that while the scatter of best-fit burst parameters over time generally exceeds the heavy error bar, they are of the same order. Further, the scatter is always significantly less than the scatter of best-fit model parameters about the trends inferred in the main text (Fig. 4) over integration positions (thin green bar). These observations further support our suggestion that distribution stability over time is not likely to affect our inference of trends in burst-parameter variation over integration positions. Finally, fitting these distributions, after applying the rescaling mentioned above (in Fig. S.3D), results in a further reduction of scatter in fit parameters (not shown).

Similarly, uncertainty generated by our instrumentation also contributes to uncertainty in identifying an experimental distribution for model fitting. The point-spread function (PSF) for our cytometer was much sharper than our experimental distributions, and its uncertainty, which we might estimate to be on the order of 10% for its relative width, would affect all distributions similarly. Theoretical analysis here suggests that uncertainty in the PSF width leads to an uncertainty in determining a transcriptional burst frequency from our fitting procedure, with the effect being quadratic. That is, uncertainties of order 10% in the relative width of the PSF typically would lead to a corresponding uncertainty of order 1% in determining the transcriptional burst frequency for each fit. As with uncertainty due to distribution drift over time, this uncertainty is on the order or smaller than that which was considered in our analysis due to our distribution processing procedure. Further, uncertainty in measuring the cytometer PSF would affect all distribution fits in the same way, scaling all inferred burst frequencies by a similar factor. Thus, even larger uncertainties in the cytometer PSF would not affect our inference of trends in burst-parameter variation over the set of clones.

As a final consideration of uncertainties in our model inference that might be due to our optimized gating procedure, which was applied to specify an experimental GFP distribution at fixed scattering measure in the main text, we re-applied our fitting procedure to the experimental distributions obtained by applying a 10% square gate in the FSC/SSC plane, as discussed in Sec. S.I.7, which is a factor of 6 narrower in each scattering dimension than the optimal gating used in the main text. The fit parameters obtained for this set of distributions are compared to those obtained in the main text, in Fig. S4. In general, best-fit model parameter for these 'narrow-gated' distributions agreed with those obtained in the main text approximately to within the 95% confidence intervals obtained by the analysis of the main text. Further, regression analysis indicates no significant change in calculated trends in burst-parameter variation with expression mean, confirming that the main results of our analysis are robust to uncertainties due our choice of distribution gating.

Overall, the above qualitative discussion, and additional systematic fits, suggest that the trends in burst parameter variation over integration position inferred in the main text are a robust result of our analysis, as are the specific values of inferred transcriptional dynamic parameters for each clone (though the later to a lesser degree).

**VIII) Considering extrinsic sources of heterogeneity**

Our analysis has assumed that expression heterogeneities in our system arise completely from the intrinsic processes represented by the model in Fig. 2A. Namely, from the probabilistic nature of the biochemical reactions involved in gene activation, transcription, and translation, at fixed values of the model parameters. Extrinsic sources of heterogeneity may be thought of as cell-cell variability in the parameters of the model. That is, the model parameters themselves may be random variables. Here we discuss features of our data and simulation results that support our intrinsic-noise-based analysis.

In earlier work, we had experimentally considered the affects of specific extrinsic factors in a similar HIV model system, including stage in cell cycle, cell size, aneuploidy, and mitotic cell division, finding little contribution to expression heterogeneity [17]. Here, we have found little correlation between cellular fluorescence and cell size, as measured by FSC, further indicating that cell growth is not a significant source of expression heterogeneity (e.g. Fig. S1.F). We found that the contribution of cell-size variability to expression heterogeneity can be

minimized by a coarse gating that includes cells whose FSC values lie in the mid 60% of the population (see Sec. S.I.3) in contrast to the narrow gating that Newman & Weissman found necessary to isolate the intrinsic component of expression variability [2], and we have noted that the large coefficient of variation of approximately 60% for the 'typical' distribution in our system is atypical when compared to the results of a number of studies that quantified expression heterogeneities from large numbers of promoters [1,16,18]. Here we continue to develop the argument that the large expression heterogeneities observed in our system more generally make an account based on extrinsic sources of heterogeneity less plausible.

Extrinsic fluctuations in processes that affect the expression of all genes should contribute similarly to expression heterogeneities from any promoter. This reasoning was applied to the large data set in [2,16], where it was suggested that extrinsic fluctuations therefore set a lower limit to the amount of expression heterogeneity the should be generated by any promoter, and therefore should only make a dominant contribution to expression heterogeneities from promoters with the sharpest expression profiles. The expression variability noted for the less 'noisy' promoters in those studies thus sets an upper bound for the relative contributions of extrinsic variability to the expression variability from an arbitrary promoter. Our measurements, on the other hand, represent the opposite end of the spectrum – the HIV promoter, for any of the sampled integrations (and especially for brighter ones), is much 'noisier' than the 'typical' eukaryotic promoter considered in the mentioned studies. We have no reason to suspect that the HIV promoter has any unique properties in terms of how it propagates extrinsic noise. We therefore argue that the increased expression noise demonstrated by the HIV promoter in our system, above the lower limit set by the class of least 'noisy' promoters that were analyzed in the mentioned studies, reflects intrinsic expression heterogeneities that are specified by features of the HIV promoter and its coupling to the host-cell environment.

From a theoretical point of view, we might consider the effects of sources of external 'noise' on the modeled processes in our system, as follows. Fluctuations in transcription rate are modeled in our system via fluctuations in promoter configuration between an active and repressed state. Any further fluctuations in this dynamic would simply be represented as a more complex stochastic process governing the gene-state dynamics and/or the inclusion of more states. We acknowledge that the representation of gene-state dynamics in our analysis is a great simplification. However, it captures the fundamental process of gene activation, and allows us to

effectively capture the expression profiles that we observe experimentally. A two-state promoter, whose dynamics are described as in our model, thus represents a parsimonious and biologically motivated account of our data. Any proposition of a more complex underlying promoter dynamic, perhaps based on hypothesized contributions of extrinsic fluctuations, would need to be directly motivated by observations that are not accounted for by the current model. For the present, there is no direct evidence to motivate consideration of a more complex promoter dynamic in our analysis.

On the other hand, even if we assume a two-state-promoter model to be correct, the transition rates between the states of our model should depend on concentrations of transcription factors that themselves fluctuate, and one could imagine that these fluctuations would generate extrinsic contributions to expression heterogeneity. To consider this possibility, we note that noise from each process in our model is filtered by the next in the chain of reactions that represent gene expression. That is, our observed protein expression heterogeneities result from much larger underlying heterogeneities in transcript distributions (see, for example, the sample distributions in Fig. 2B of the main text). In particular, if we consider the coefficient of variation as a measure of expression heterogeneity, then in the bursting regime, using eqs. 14 and 15, we find that the coefficient of variation for the protein and transcript distributions are related as:

$$CV_P^{\;2} = \frac{\sigma_P^{\;2}}{\mu_P^2} \sim \frac{\kappa_p^{\;-}}{\kappa_p^{\;-} + \kappa_t^{\;-}} CV_T^{\;2} = 0.2 CV_T^{\;2} \qquad (16)$$

where we have inserted our experimentally measured value for the ratio of protein and transcript degradation rates in the last equality.

Similarly, if we consider the coefficient of variation of the transcription-rate distribution over the population (the transcription rate dynamics is determined by the gene-state dynamics, with values $\kappa_t^+$ or 0 for the transcription rate, depending if the gene is in the inactive or active state) we find

$$CV_G^2 = \kappa_r / \kappa_a \qquad (17)$$

which becomes large in the bursting regime (where $\kappa_r >> \kappa_a$). The coefficient of variation for the gene-state (transcription rate) is related to the coefficient of variation for the transcript distribution according to

$$CV_G^2 = \left(\frac{b}{b+1}\right)\left(\frac{\kappa_r}{\kappa_t^-}\right) CV_T^2 \qquad (18)$$

where the bursting regime has again been assumed. The first factor on the RHS becomes small only for burst sizes of order 1 or smaller, i.e. when transcripts are effectively produced one at a time and their distribution becomes indistinguishable from what would result from production in a continuous Poisson process. For large burst sizes, this factor approaches 1, and the second factor becomes large in the bursting regime.

Thus, in the bursting regime, for burst sizes that are not small

$$CV_G^2 >> CV_T^2 > CV_P^2 . \qquad (19)$$

Therefore, for our experimental distributions, our analysis predicts that the 'noisy' protein expression distributions that we measured are generated by yet 'noisier' underlying transcript distributions, which are in turn generated by yet 'noisier' underlying gene-state dynamics. And this is generally the case for a cascade of stochastic processes of the type that we are considering here. For noise in an upstream process to make a significant contribution to heterogeneity in the distribution of a down-stream component, the upstream process should be significantly noisier (and/or slower) than the down-stream process to which it couples. Of course the situation becomes more complicated if feedback is considered, but because our reporter system only produces GFP, we do not expect any feedback effects.

We thus conclude that for fluctuations in upstream components – such as transcription factors that directly couple to the LTR – to significantly contribute the expression heterogeneity from the LTR, their dynamics should be noisier than the predicted gene-state dynamics of the LTR. But the gene-state dynamics of the LTR are necessarily noisier than its protein expression dynamics, which in turn are likely noisier than the protein expression dynamics of most cellular proteins, since the LTR is noisier than the vast majority of promoters considered in other studies, as discussed above. Thus, we suggest that it is unlikely that fluctuations in the expression of upstream proteins that interact with the LTR significantly affect the expression distributions that we observe from the LTR.

We make one final comment concerning extrinsic fluctuations in our system. For the moment it is left as an open question specifically which processes are represented by the gene-state dynamics of our model, though we have made several suggestions in the Discussion section of the main text, and we have further suggested that the process of gene activation and inactivation in our model might be part of a larger-scale chromatin/genomic dynamic. Such a dynamic would affect the expression of multiple genes simultaneously. Since a common probe

for extrinsic fluctuations is the use of two-reporter systems [19], one would thus expect to find expression correlations between two independent reporters in such a system. It is still an open and important problem in eukaryotic transcriptional biology, to understand the large-scale genomic and nuclear dynamics that affect the expression dynamics of multiple genes simultaneously, and more specifically, to identify and distinguish between contributions of extrinsic and intrinsic sources of expression noise in such a system where these two contributions may be highly integrated.

**REFERENCES**


1. Sigal A, Milo R, Cohen A, Geva-Zatorsky N, Klein Y, et al. (2006) Variability and memory of protein levels in human cells. Nature 444: 643-646.
2. Newman JR, Ghaemmaghami S, Ihmels J, Breslow DK, Noble M, et al. (2006) Single-cell proteomic analysis of S. cerevisiae reveals the architecture of biological noise. Nature 441: 840-846.
3. Gardiner CW (2004) Handbook of stochastic methods : for physics, chemistry, and the natural sciences. Berlin ; New York: Springer. xvii, 415 p. p.
4. Gillespie DT (2007) Stochastic simulation of chemical kinetics. Annu Rev Phys Chem 58: 35-55.
5. Peccoud JaY, B. (1995) Markovian Modeling of Gene-Product Synthesis  Theor Pop Biol 48: 13.
6. Schultz D, Onuchic JN, Wolynes PG (2007) Understanding stochastic simulations of the smallest genetic networks. J Chem Phys 126: 245102.
7. Hornos JE, Schultz D, Innocentini GC, Wang J, Walczak AM, et al. (2005) Self-regulating gene: an exact solution. Phys Rev E Stat Nonlin Soft Matter Phys 72: 051907.
8. Munsky B, Khammash M (2006) The finite state projection algorithm for the solution of the chemical master equation. J Chem Phys 124: 044104.
9. Kampen NGv (1992) Stochastic processes in physics and chemistry. Amsterdam ; New York: North-Holland. xiv, 465 p. p.
10. Griffith M, Courtney T, Peccoud J, Sanders WH (2006) Dynamic partitioning for hybrid simulation of the bistable HIV-1 transactivation network. Bioinformatics 22: 2782-2789.
11. Cao Y, Gillespie DT, Petzold LR (2007) Adaptive explicit-implicit tau-leaping method with automatic tau selection. J Chem Phys 126: 224101.
12. Chevalier MW, El-Samad H (2009) A rigorous framework for multiscale simulation of stochastic cellular networks. J Chem Phys 131: 054102.
13. Elf J, Lotstedt P, Sjoberg P. Problems of High Dimension in Molecular Biology. Proceedings of the 19th GAMM-Seminar; 2003; Leipzig. Max-Planck-Institute for Mathematics in the Sciences. pp. 21-30.
14. Press WH (1997) Numerical recipes in C : the art of scientific computing. Cambridge ; New York: Cambridge University Press. xxvi, 994 p. p.
15. Raser JM, O'Shea EK (2004) Control of stochasticity in eukaryotic gene expression. Science 304: 1811-1814.
16. Bar-Even A, Paulsson J, Maheshri N, Carmi M, O'Shea E, et al. (2006) Noise in protein expression scales with natural protein abundance. Nat Genet 38: 636-643.
17. Weinberger LS, Burnett JC, Toettcher JE, Arkin AP, Schaffer DV (2005) Stochastic gene expression in a lentiviral positive-feedback loop: HIV-1 Tat fluctuations drive phenotypic diversity. Cell 122: 169-182.
18. Newman JR, Weissman JS (2006) Systems biology: many things from one. Nature 444: 561-562.
19. Elowitz MB, Levine AJ, Siggia ED, Swain PS (2002) Stochastic gene expression in a single cell. Science 297: 1183-1186.
